# Faecalibacterium prausnitzii prevents age-related heart failure by suppressing ferroptosis in cardiomyocytes through butyrate-mediated LCN2 regulation

**DOI:** 10.1080/19490976.2025.2505119

**Published:** 2025-05-13

**Authors:** Yun Zhang, Ying Wei, Xuejie Han, Ling Shi, Hui Yu, Xuanrui Ji, Yunlong Gao, Qianhui Gao, Linwei Zhang, Yu Duan, Wenpeng Li, Yue Yuan, Jing Shi, Liang Cheng, Yue Li

**Affiliations:** a Department of Cardiology, The First Affiliated Hospital, Harbin Medical University, Harbin, China; b NHC Key Laboratory of Cell Transplantation, The First Affiliated Hospital of Harbin Medical University, Harbin, Heilongjiang, China; c Key Laboratory of Cardiac Diseases and Heart Failure, Harbin Medical University, Harbin, China; d State Key Laboratory of Frigid Zone Cardiovascular Disease, Harbin Medical University, Harbin, Heilongjiang, China; e College of Bioinformatics Science and Technology, Harbin Medical University, Harbin, Heilongjiang, China

**Keywords:** Heart failure, aging, *F. prausnitzii*, butyrate, ferroptosis

## Abstract

Aging is a primary driver of the escalating prevalence of heart failure (HF). Age-associated gut microbiota dysbiosis has been implicated in various age-related diseases, yet its role in age-related HF remains largely unexplored. In this study, we sought to explore the potential link between age-related gut microbiota alterations and HF in the elderly. We analyzed a publicly available single-cell sequencing dataset, which revealed markedly increased ferroptosis activity in cardiac myocytes of elderly individuals compared to their younger counterparts. Notably, treatment with the ferroptosis inhibitor, ferrostatin-1, mitigated cardiac ferroptosis and prevented cardiac dysfunction in aging rats. Furthermore, fecal microbiota transplantation from elderly HF patients significantly increased cardiac ferroptosis activity and induced cardiac dysfunction in healthy recipient rats. Integrated 16S rRNA sequencing and PCR quantification revealed a marked depletion of *Faecalibacterium prausnitzii* (*F. prausnitzii*) in elderly individuals, with a more pronounced decline in elderly patients with HF. Oral administration of *F. prausnitzii* or its metabolite butyrate effectively attenuated age-related HF through inhibiting ferroptosis. Additionally, gene-editing techniques were employed to generate *F. prausnitzii* BCoAT mutant deficient in butyrate production. Intriguingly, the protective effect was lost in the butyrate-deficient *F. prausnitzii* strain. Mechanistically, butyrate reduced intracellular iron accumulation and suppressed ferroptosis by downregulating LCN2 expression in senescent cardiomyocytes. Our findings highlight the critical role of aged microbiota-induced ferroptosis in HF and propose *F. prausnitzii* or butyrate may serve as potential targets for the prevention and treatment of age-related HF.

## Introduction

Heart failure (HF) is a life-threatening clinical syndrome characterized by substantial morbidity and mortality, with its prevalence steadily rising worldwide.^1^ Advanced age has emerged as a predominant risk factor for HF, with its incidence escalating significantly as individuals age. Epidemiological studies reveal HF prevalence escalates from 1% to 3% in the general adult population to 8% in adults aged 65–74 y, surpassing 16% in those over 75.^2,3^ Consequently, HF has become a leading cause of mortality among the elderly. As the global population ages, age-related HF poses a significant challenge in modern healthcare systems. Despite the urgent need for effective therapeutic strategies, the precise mechanisms driving age-related HF poorly understood, highlighting a critical gap in our knowledge.

The intricate interplay between the gut microbiota and cardiovascular diseases has attracted considerable research attention.^4–6^ As the composition and function of gut microbiota evolve with host aging, they exert detrimental effects, such as compromised gut epithelial barrier integrity and heightened low-grade systemic inflammation. This age-related gut microbiota dysbiosis increases the host’s vulnerability to chronic conditions associated with aging.^7–9^ Our previous studies demonstrated that age-related microbiota dysbiosis drives atrial fibrillation via NLRP3-inflammasome activation.^10^ However, the role of age-related microbiota dysbiosis in HF remains a critical knowledge gap that warrants further elucidation.

Ferroptosis, an iron-dependent programmed cell death driven by lipid peroxidation,^11^ has emerged as a significant factor in age-related diseases, including Alzheimer’s disease, Parkinson’s disease, and osteoporosis.^12–14^ Recent advances implicate ferroptotic cell death in ischemic cardiomyopathy, myocardial infarction, and reperfusion injury.^15–17^ Importantly, microbial-derived metabolites are increasingly recognized as key regulators of ferroptotic pathways.^18,19^ For example, *Lactobacillus vaginalis* has been shown to inhibit Fdps-mediated hepatocyte ferroptosis.^20^ Despite these advances, mechanistic connections between age-related gut dysbiosis, ferroptosis, and HF progression remain unclear.

In this study, we establish gut microbiota dysbiosis as a causal driver of age-related HF through ferroptosis modulation. Using single-cell sequencing analysis of publicly available datasets, we identified ferroptosis as a key driver of cardiac dysfunction in aging, a finding further validated through FMT experiments. We demonstrated that aged microbiota exacerbates ferroptosis and induces HF in young rats, while oral supplementation with *F. prausnitzii* confers cardioprotection. Notably, a *F. prausnitzii* mutant deficient in BCoAT, which is unable to produce butyrate, failed to mitigate the pro-HF effects of aged microbiota, highlighting the critical role of butyrate in this process. These findings not only identify *F. prausnitzii* supplementation and butyrate administration as potential therapeutic strategies but also elucidate their mechanistic role in modulating ferroptosis within the gut-heart axis during aging. Our study offers innovative microbiota-based strategies for both prevention and treatment of age-related HF, bridging a critical gap in cardiovascular research.

## Materials and methods

### Human samples

The use of human sample complies with the Declaration of Helsinki and was approved by the Ethics Committee of the First Affiliated Hospital of Harbin Medical University (Ethical approval number: IRB-AF/SC-04/02.0). The participants all provided a written informed consent. The animal experiments were conducted in accordance with the Guide for the Care and Use of Laboratory Animals and approved by the Institutional Animal Care and Use Committee at Harbin Medical University. This study included 40 elderly healthy individuals and 40 elderly heart failure patients (aged 65–80) admitted to the Department of Cardiovascular Medicine at the First Affiliated Hospital of Harbin Medical University from July 2022 to December 2022. Exclusion criteria encompassed conditions such as hyperthyroidism, autoimmune diseases, malignancies, severe systemic infectious diseases, hematologic disorders, and a history of organ transplantation; severe arrhythmia, acute myocardial infarction, acute myocarditis, endocarditis, etc.; severe liver or kidney dysfunction; concomitant history of cancer or other gastrointestinal inflammatory diseases; use of antibiotics or probiotics in the 3 months prior to sampling; occurrence of upper respiratory tract infections in the month preceding sampling.

### Experimental animals

The animal experiments in this study were performed in accordance with the Guide for the Care and Use of Laboratory Animals and approved by the Institutional Animal Care and Use Committee at the Harbin Medical University (Ethical approval number: 2022118). All animal procedures performed also conform to the guidelines from Directive 2010/63/EU of the European Parliament on the protection of animals used for scientific purposes or the current NIH guidelines. Male SD rats (200–250 g) were purchased from Beijing Vital River Laboratory Animal Technology Co, Ltd (Beijing, China) and raised at the Experimental Animal Center of Harbin Medical University (Harbin, China). Only male rats were used in the study because females are markedly resistant to iron overload-induced cardiomyopathy.^21^ The animals were kept in cages with light/dark cycles of 12 h and fed with food and water available ad libitum in SPF conditions. After 1 week of adaptive feeding, rats were used for experiments. At the end of the experiments, all rats were anesthetized with pentobarbital sodium and euthanized via cervical dislocation. Tissue samples were collected immediately for further analysis. The neonatal Sprague-Daw rats were anesthetized by isoflurane inhalation and sacrificed by cervical dislocation, and these animals were used for collection of primary cells.

To test whether ferroptosis is the key mechanism of age-related heart failure, 18 months old rats were randomly receiving vehicle or ferrostatin-1 (MedChemExpress, HY-100579) at a dose of 0.8 mg/kg through intraperitoneal injection once weekly for 4 months.

To determine whether aged microbiota promotes cardiac dysfunction, rats were initially subjected to a one-week antibiotic treatment to deplete their intestinal microbiota. Subsequently, the rats were randomly divided into two experimental groups: one group received fecal microbiota transplantation (FMT) derived from young donors (2–3 months old), while the other group received FMT from aged donors (22–24 months old). FMT was administered daily over a 12-week period to assess its effects on cardiac function.

To assess whether microbiota from elderly HF patients promotes HF in rats through regulation of ferroptosis, fecal samples were collected from healthy control subjects and elderly HF patients. Rats underwent one-week antibiotic treatment to deplete their intestinal microbiota and were subsequently randomized into three groups. The first group received microbiota from healthy control subjects (healthy control group), while the other two groups received microbiota from elderly heart failure (HF) patients. Among these, one group was intraperitoneally injected with the ferroptosis inhibitor ferrostatin-1 (0.8 mg/kg) once weekly for 12 weeks (elderly HF+Fer-1 group), and the other group received an equivalent volume of saline (elderly HF group).

To investigate the role of *F. prausnitzii* in age-related HF, aging was induced in rats via daily subcutaneous injection of D-galactose (150 mg/kg/day) for 8 weeks. Rats were randomly divided into three groups: the Aging group received D-galactose via subcutaneous injection and PBS via oral gavage; the Aging+FP group received D-galactose via subcutaneous injection combined with oral gavage of *F. prausnitzii* (1 × 10^9^ CFU, three times per week); and the Control group received saline via subcutaneous injection (volume-matched to D-galactose). Furthermore, we collected fecal samples from elderly HF patients for microbiota transplantation. Rats received a one-week antibiotic treatment for depletion of intestinal microbiota. Thereafter, the rats were randomly divided into two groups. One group received microbiota transplantation from elderly patients, concurrent with a vehicle treatment (Aging-FMT+Veh group), while another group received microbiota transplantation from elderly patients combined with *F. prausnitzii* (Aging-FMT+FP group). Rats in the Aging-FMT+FP group were orally gavaged with 1 mL *F. prausnitzii* suspension (1 × 10^9^ CFU/mL), three times per week for 12 weeks.

To assess the role of butyrate in age-related HF, rats administered with D-galactose (150 mg/kg/day) via subcutaneous injection were randomly divided into two groups, one receiving water containing butyrate sodium (1% w/w) for 8 weeks (Aging+Butyrate group) and another receiving ordinary water (Aging+Veh group).

To demonstrate the cardioprotective effect of *F. prausnitzii* via butyrate, we generated a BCoAT mutant of the *F. Prausnitzii* strain (*F.P*ΔBCoAT) that was unable to generate butyrate using the homologous recombination technique. Rats were divided into three groups, aging group was D-galactose-induced aging rats, aging+*F.P*ΔBCoAT group was aging rats treated with *F.P*ΔBCoAT, and aging+*F.P*ΔBCoAT+butyrate group was treated with *F. P*ΔBCoAT combined with butyrate. Rats were gavaged with 1 × 10^9^ CFU *F.P*ΔBCoAT, three times a week for 8 weeks. Butyrate was administrated with water containing butyrate sodium (1% w/w) for 8 weeks.

### Echocardiography

The rats were anesthetized via intraperitoneal injection of 1% sodium pentobarbital (30 mg/kg). Following anesthesia, echocardiography was performed on rats to assess cardiac structure and function using a Vivid 7 echo machine (GE Healthcare, Milwaukee, WI, USA) with two-dimensional M-mode analysis. The cardiac ejection fraction (EF), and fractional shortening (FS) were measured over at least five consecutive cardiac cycles.

### Fecal microbiota transplantation

Fecal samples were collected aseptically, and each 120 mg fecal sample was dissolved in 1 mL of sterile phosphate-buffered saline (PBS) and thoroughly homogenized to ensure a uniform suspension. The homogenized solution was then centrifuged at 800 × g for 3 min to remove large particulate matter and debris. The bacteria-enriched supernatant was carefully collected, and glycerol was added to the bacterial suspension to achieve a final concentration of 20% (v/v), and the samples were stored at −80℃. To promote re-colonization of transplanted gut microbiota, rats were treated with antibiotic cocktails containing vancomycin (100 mg/kg), ampicillin (200 mg/kg), neomycin (200 mg/kg), and metronidazole (200 mg/kg) via oral gavage for 1 week prior to FMT to deplete their endogenous microbiota. Thereafter, a single oral gavage (1 mL) was administered weekly for 12 weeks to facilitate the re-colonization of the transplanted gut microbiota.

### Histopathology

Whole hearts and ventricular tissue from the rats were collected, fixed in 4% paraformaldehyde, imbedded in paraffin, and serially sectioned at 4 µm thickness. Subsequently, the tissues were stained with HE and Masson trichrome staining. Fibrotic area was quantified using ImageJ software and the collagen volume fraction was calculated as collagen area/total area × 100%.

### *Cultivation and inactivation* F. prausnitzii

F. *prausnitzii* (MSD 17,677) was purchased from Shanghai Ningbo Mingzhou Technology Co., Ltd. It was routinely cultured in Modified Reinforced Clostridial Broth Medium (MD039B, Shandong Toupu Bioengineering Co., Ltd, China) at 37 ℃ in an anaerobic chamber using gas mix consisting of 10% hydrogen, 10% carbon dioxide and 80% nitrogen for 24–48 h. For *F. prausnitzii* transplantation, bacteria were collected by centrifugation at 3500 rpm/min for 10 min and resuspended in sterilized saline with 20% (v/v) glycerol. Rats were treated with 1 × 10^9^ colony-forming units (CFU) of live *F. prausnitzii* (1 mL) or *F. P*ΔBCoAT (1 mL) by gavage, three times a week. Inactivated *F. prausnitzii* was prepared by heating bacteria at 95℃ for 15 min in a water bath.

### *Construction of the BCoAT knockout strain in* Faecalibacterium prausnitzii

The butyryl-CoA:acetate CoA-transferase gene (BCoAT) in *Faecalibacterium prausnitzii* was disrupted via homologous recombination. Upstream (756 bp, primers Up-F/R) and downstream (665 bp, primers Dw-F/R) regions of BCoAT were PCR-amplified and fused with the ermB resistance cassette from pGhost9. The fragment was cloned into suicide vector pMTL83151 using NEBuilder HiFi Assembly with NotI/HindIII sites. The recombinant plasmid was transformed into *E. coli* WM3064 and conjugated with *F. prausnitzii* grown in reinforced clostridial medium (RCM). After 24-h anaerobic mating on DAP-supplemented RCM agar, transconjugants were selected with 5 μg/mL erythromycin. Three rounds of nonselective subculture promoted plasmid excision, followed by validation on erythromycin/chloramphenicol plates. Single clones were selected, and mutation of the targeted gene was validated by colony PCR. The butyric acid production ability of mutant *F. prausnitzii* was evaluated by GC-MS/MS.

### Culture of primary rat cardiomyocytes and treatment

The primary rat cardiomyocytes were obtained from hearts of neonatal Sprague-Daw rats (1–3 d old). Briefly, the neonatal Sprague-Daw rats were anesthetized with isoflurane inhalation and sacrificed by cervical dislocation. Following this, the hearts was separated under aseptic conditions and cut to small pieces. Thereafter, the tissue was digested in 0.25% tryspin with gently shaking. All digestive fluid was collected in DMEM with 10% fetal bovine serum, centrifuged at 1200 rpm for 5 minutes. The cells were resuspended in DMEM containing 10% FBS, 1% penicillin and streptomycin, which was inoculated in culture dish for 90 minutes. The pre-seeding medium containing cardiomyocytes were seeded in new six-well plates, and stayed at 37°C in a incubator with 5% CO_2_.

Upon reaching a fusion rate of 70%–80% and exhibiting good condition, the treatment was initiated. The optimal concentration and duration for constructing an senescent cardiomyocyte with H_2_O_2_ were determined to be 20 μmol/L for 6 h, while sodium butyrate treatment was set at 5 mmol/L for 42 h. The cell culture medium was first replaced with serum-free culture medium, then after 6 h of H_2_O_2_ exposure, it was switched to fresh culture medium before the addition of sodium butyrate or *F. prausnitzii*, followed by 42 h of subsequent testing.

### *Co-culture of* F. prausnitzii *and cardiomyocytes*

Primary cardiomyocytes and *F. prausnitzii* were pre-cultured independently. Our optimized protocol ensures *F. prausnitzii* viability while maintaining cardiomyocyte physiology through a two-phase oxygen-controlled workflow: (1) Phase 1 (0–6 h): Co-culture under strict anaerobic conditions (without O₂) using a 0.4 μm PET Transwell system, allowing metabolite exchange while preventing bacterial-cell contact; (2) Phase 2 (6–48 h): Cardiomyocytes transferred to normoxia (21% O₂) for functional assays, with bacterial compartments removed to eliminate hypoxia artifacts. For *F. prausnitzii* supernatant intervention experiment, the *F. prausnitzii* cultures were centrifuged at 5000 g at 4℃ for 15 min, followed by collection and filtration of the supernatants through a 0.22 µm polyethersulfone membrane. After mixing the supernatant and cell culture medium at a 1:10 ratio, 1 mL of the mixture was added to each well of a six-well plate for 6 h. Subsequently, the cardiomyocytes were washed twice with PBS and further cultured for 42 h.

### Measurement of GSH

The plasma and ventricular tissue were collected for detection of GSH by ELISA according to the manufacturer’s instructions (MEIMIAN, MM-0602R1).

### MTT assay

The cell viability was determined using the MTT Cell Proliferation Assay (Beyotime, Shanghai, China). Briefly, cells were seeded in 96-well plates. After treatment, 10 μL MTT was added to the cells in phenol red-free culture medium and incubated for 4 h at 37°C. Then, DMSO was added to the cells, and the absorbance was measured by a microplate reader (Thermo, Massachusetts, USA).

### Measurement of mitochondrial membrane potential

The mitochondrial membrane potential (MMP) was determined with JC-1 staining kit (Beyotime) in accordance to the manufacturer’s instructions. Briefly, the primary rat cardiomyocytes were incubated with JC-1 staining solution (5 pg/mL) at 37°C for 20 min and rinsed with JC-1 staining buffer. Images were observed through fluorescence microscopy (Zeiss, Jena, Germany). The intensity ratio of green fluorescence vs. red fluorescence was calculated using the Image J software. Mitochondrial depolarization is indicated by an increase in the green/red fluorescence intensity ratio.

### Intracellular reactive oxygen species (ROS) detection

Intracellular ROS levels were determined using Dihydroethidium (S0063, Beyotime). In brief, primary rat cardiomyocytes cultured in 6-well plates were incubated with 5 μM Dihydroethidium in serum-free medium at 37℃ for 30 min in the dark. After washing, the cells were analyzed by immunofluorescence microscope (Zeiss, Jena, Germany). The intensity fluorescence was calculated using the Image J software.

### Transmission electron microscopy

Samples of myocardium were quickly removed from the left ventricle of rats, and immediately fixed in 3% glutaraldehyde. Once completed, embedded and cut, and viewed using a Tecnai 10 (100 kV) transmission electron microscope (FEI).

### Western blot

The total protein samples were extracted from tissues of rats or cardiomyocytes. Briefly, approximately 30 μg proteins were resolved in 8%~12% SDS-PAGE, and transferred to PVDF membranes (Millipore, Billerica, MA, USA). The samples were incubated with primary antibodies for Tf (1:500, 17435–1-AP, Proteintech), FTH1 (1:500, bs-5907 R, Bioss), GPX4 (1:500, ab125066, Abcam), anti-LCN2 (1:500, ab216462, Abcam) and GAPDH (1:10000, ab 128,915, Abcam) at 4℃ overnight. After washing, the membrane was incubated with anti-IgG horseradish peroxidase-conjugated secondary antibody. The membranes were exposed to ECL buffer and detected by ChemiDoc XRS gel documentation system (Bio-Rad, Hercules, CA, USA).

### *qRT-PCR* *analysis of the relative abundance of* F. prausnitzii

Fecal samples were collected from patients (Elderly healthy and Elderly HF) and rats (Control group, Aging group, Aging+FP group, Aging-FMT+Veh group and Aging-FMT+FP group), extracted bacterial DNA, and quantified the relative abundance of *F. Prausnitzii* using quantitative real-time PCR (qRT-PCR). The relative abundance was determined by comparing the Ct values of *F. Prausnitzii* to 16S rRNA gene in the same sample, following the 2^-ΔΔCt method.

### scRNA-seq data processing

The data processing pipeline included integration, batch effect correction, normalization, dimensionality reduction, and clustering. Dimensionality reduction was performed using Uniform Manifold Approximation and Projection (UMAP). The grouping of samples was based on age information from the metadata. Cell type proportions were calculated for each group, with a specific focus on ventricular cardiac myocytes for subsequent re-clustering and differentially expressed genes (DEGs) analysis. Ventricular cardiac myocytes were further clustered into four distinct subgroups using UMAP, with the top three marker genes for each cluster identified. DEG analysis was conducted using ‘scanpy.tl.rank_genes_groups’ (with method = ‘Wilcoxon’ and reference=‘Young’). Genes with adjusted P-value <0.05 and logFC >0.25 were considered significantly differentially expressed. Kyoto Encyclopedia of Genes and Genomes (KEGG) enrichment analysis was performed with normalized and log1p-transformed gene expression data to compare elderly group and young group via GSEApy (version 1.1.3).

### 16S rRNA amplicon sequencing data analysis

Alpha diversity indexes including Chao1 and Shannon and beta diversity were both calculated and visualized using the R package MicrobiotaProcess (version 1.19.0). The relative abundance at the genus level was calculated, and the log2FC values of the Elderly group compared to the Young group were computed. A rank-sum test was used to compare the two groups. The result was visualized using the R package ggplot2 (version 3.5.1).

### Transcriptome analysis

The principal component analysis (PCA) of the two groups was performed using the vegan (v2.6–10). Differential expression analysis was carried out using DESeq2 (v1.16.0), with genes having a p-value <0.05 and |logFC| >2 identified as differentially expressed. The top 20 up-regulated genes were visualized using pheatmap (v1.0.12). Gene Ontology (GO) enrichment analysis of the differentially expressed genes was performed using clusterProfiler (v4.14.4).

### Statistical analysis

Statistical analysis was performed using GraphPad Prism 8.0 software (GraphPad Software, Inc., La Jolla, CA). Shapiro–Wilk test was used for normality test. Continuous variables were expressed as mean ± standard error of mean (SEM) or median and interquartile range. Categorical variables were represented as numbers and percentages. Two- group comparisons were performed using non-paired Student’s t-test or Mann–Whitney U test for continuous variables. Variables with more than two groups were analyzed by one-way ANOVA, followed by Tukey tests. Chi-square test or Fisher’s exact test was used for comparisons of categorical variables. Two-tailed and *p* < 0.05 were considered statistically significant.

## Results

### Ferroptosis in cardiomyocytes as a driver of age-related heart failure

**Figure 1.**
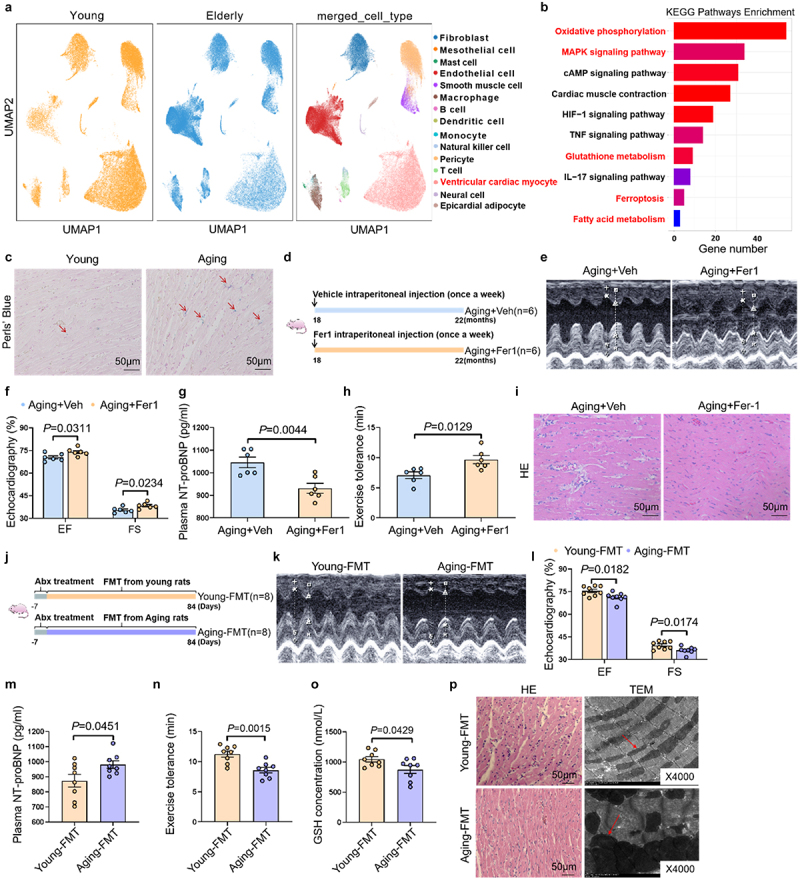
Age-related gut microbiota increases cardiac ferroptosis and promotes heart failure. (a) UMAP plot of single-cell RNA sequencing data showing the clustering of different cell populations in heart tissue of young and elderly individuals. (b) KEGG pathway enrichment analysis showed the pathways significantly associated with gene expression changes in ventricular cardiac myocytes. (c) Representative image of Perls’ blue staining in the left ventricle of young and aging rats. (d) Schematic illustration of the experimental design for ferroptosis inhibitor intervention in aging rats. 18 months old rats were randomly subjected to receive vehicle or ferroptosis inhibitor (ferrostatin-1, 0.8 mg/kg, intraperitoneal injection), once a week for 12 weeks. (e) Representative M-mode images of left ventricular wall motion in the hearts of rats from aging+Vehicle group and aging+Fer-1 group. (f) Quantification of cardiac function parameters, cardiac ejection fraction (EF) and cardiac fraction shortening (FS) of rats from aging+Vehicle group and aging+Fer-1 group (*n* = 6 per group). (g) Quantitative analysis of plasma NT-proBNP levels in rats from aging+Vehicle group and aging+Fer-1 group (*n* = 6 per group). (h) The exercise tolerance in rats (*n* = 6 per group). (i) Representative images of HE staining of the left ventricle of hearts in rats. (j) Schematic illustration of the experimental design for microbial transplantation experiments. The feceal samples were collected from young and aging rats for microbial transplantation experiments. Before microbial transplantation, rats were treated with antibiotics for 1 week to deplete gut microbiota. Afterwards, microbiota-depleted rats received microbiota from young rats, or aging rats through gavage, once a day for 12 weeks. (k) Representative M-mode images of left ventricular wall motion in the hearts of rats. (l) The statistical data of cardiac ejection fraction (EF) and fraction shortening (FS) of rats (*n* = 8 per group). (m) Quantitative analysis of plasma NT-proBNP levels in rats (*n* = 8 per group). (n) The exercise tolerance in rats (*n* = 6 per group). (o) Quantitative analysis of GSH levels in rats (*n* = 8 per group). (p) Representative images of HE staining of the left ventricle, and representative transmission electron microscopy images of mitochondria in the left ventricle of rats.

To delineate the mechanisms underlying cardiac aging, we performed integrated reanalysis of publicly available single-cell RNA sequencing datasets (ERP123138) from cardiac tissues of three young and three elderly individuals.^22^ Uniform manifold approximation and projection (UMAP) clustering revealed distinct differences in cellular compositions between the two groups, primarily characterized by a significant reduction in cardiomyocyte proportion in elderly individuals (Figure 1(a), Figure S1A). KEGG pathway enrichment analysis demonstrated significant enrichment of ferroptosis-related pathways in cardiomyocyte subclusters of elderly individuals (Figure 1(b)). Unsupervised clustering stratified ventricular cardiomyocytes into four transcriptionally distinct subpopulations (cardiac myocyte_sub_1 to cardiac myocyte_sub_4), with cardiac myocyte_sub_2 exhibiting progressive age-dependent expansion (Figure S1B). Notably, its marker genes (FTL and FTH1) are known to promote ferroptosis (Figure S1C). Consistent with these findings, aged rats exhibited substantial iron accumulation in cardiac tissues compared to young controls (Figure 1(c)). To further validate ferroptosis involvement in age-related HF, we administered the specific ferroptosis inhibitor ferrostatin-1 (Fer-1, 0.8 mg/kg via intraperitoneal injection) to 18-month-old Sprague-Dawley rats for 4 months (Figure 1(d)). Fer-1 treatment significantly ameliorated cardiac function, evidenced by an increase in left ventricular ejection fraction (EF) and fractional shortening (FS), along with reductions in NT-proBNP levels and enhanced exercise tolerance (Figure 1(e–h)). Histopathological analysis demonstrated ferroptosis inhibitor notably reversed the structural disorganization observed in the hearts of aged rats (Figure 1(i)). At the molecular level, Fer-1 caused an increase in the expression of cardiac GPX4 and FTH1, alongside a decrease in Tf expression (Figure S2A-B). Together, these findings highlight ferroptosis as a critical mechanism driving age-related HF.

### Age-related gut microbiota dysbiosis induces cardiac dysfunction in young rats

Emerging evidence implicates gut dysbiosis in age-related pathologies, including our prior discovery linking microbial shifts to atrial fibrillation in aging.^10,23–26^ To test its role in heart failure,

we performed fecal microbiota transplantation (FMT) in young rats (8-week-old) using fecal samples from either 24-month-old (Aging-FMT) or 8-week-old (Young-FMT) counterparts over a 12-week period (Figure 1(j)). Notably, transplantation of microbiota from aging rats induced a significant reduction in cardiac ejection fraction (EF) and fraction shortening (FS) (Figure 1(k–l)), accompanied by elevated plasma NT-proBNP levels and reduced exercise tolerance (Figure 1(m–n)). Rats in the Aging-FMT group also exhibited exacerbated cardiac structural disarray, alongside heightened ferroptosis, as indicated by increased membranous density, reduced mitochondrial cristae, and elevated glutathione (GSH) levels, compared to those in the Young-FMT group (Figure 1(o–p)). Additionally, rats in the Aging-FMT group displayed higher expression of Tf and lower expression of FTH1 and GPX4 in cardiac tissues than that in the Young-FMT group (Figure S3A-B). Taken together, these findings suggest that age-related gut microbiota dysbiosis promotes ferroptosis and contributes to cardiac dysfunction in rats.

To bridge rodent findings to human pathophysiology, we established humanized rats through FMT from elderly HF patients (Figure S4A). Notably, Recipient rats receiving elderly HF patients microbiota developed significant cardiac impairment, evidenced by a reduction in EF and FS, elevated plasma NT-proBNP levels, and decreased exercise tolerance. However, Fer-1 treatment restored cardiac function (Figure S4B-E). Rats receiving microbiota from elderly HF patients exhibited structural abnormalities, which were abrogated by Fer-1 treatment (Figure S4F). Furthermore, Fer-1 treatment reduced Tf expression, and increased FTH1 and GPX4 expression (Figure S4G-H). These findings indicate that aged-related microbiota dysbiosis promotes cardiac dysfunction by modulating ferroptosis.

### *Oral supplementation of* F. prausnitzii prevents rats from age-related heart failure

**Figure 2.**
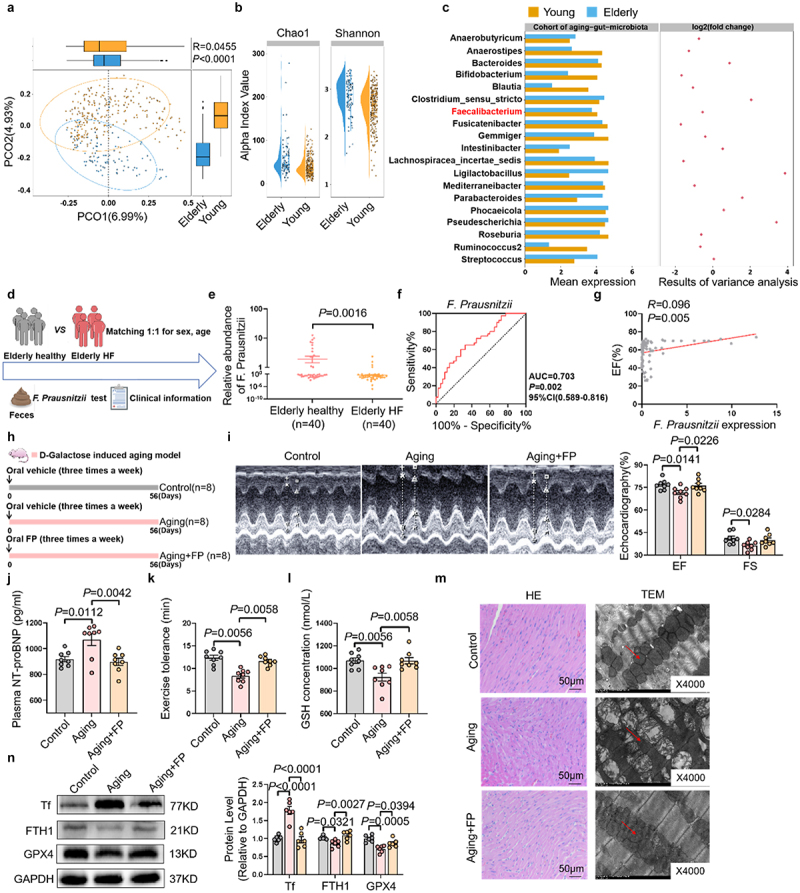
*F. prausnitzii* inhibits cardiac ferroptosis and prevents the development of age-related heart failure. (a) The PCoA showed that the gut taxonomic composition was significantly different between young (*n* = 250) and elderly individuals (*n* = 98). (b) The alpha diversity of microbial community in young and elderly individuals. (c) Secondary analysis of previously published 16S rRNA sequencing data showed the gut microbiota composition between young and elderly individuals (DRA004160-). (d) Schematic diagram of the experiment: fecal samples were collected from healthy elderly individuals and age- and sex-matched elderly heart failure patients to analyze the abundance of *F. prausnitzii*. Clinical information was also collected. (e) The relative abundance of *F. prausnitzii* in elderly healthy individuals and patients with elderly HF (*n* = 40 per group). (f) ROC curve analysis of *F. prausnitzii* abundance for the diagnosis of heart failure, showing an AUC of 0.703 (*P* = 0.002, 95% CI: 0.589–0.816). (g) Correlation analysis between *F. prausnitzii* abundance and ejection fraction (EF%), showing a positive correlation (*R* = 0.096, *P* = 0.005). (h) Schematic illustration of the experimental design. We established an aging rat model with D-galactose (150 mg/kg/day) via subcutaneous injection. The rats were randomly divided into a control group, a D-galactose-induced aging rats group, and a D-galactose-induced aging rats supplemented with *F. prausnitzii* group. (i) Representative M-mode images of left ventricular wall motion in the hearts, and the statistical data of cardiac ejection fraction (EF) and fraction shortening (FS) of rats (*n* = 8 per group). (j) Quantitative analysis of plasma NT-proBNP levels in rats (*n* = 8 per group). (k) The exercise tolerance in rats (*n* = 8 per group). (l) The levels of GSH in rats (*n* = 8 per group). (m) Representative images of HE staining, and representative transmission electron microscopy images of mitochondria in the hearts in rats. (n) Representative bands and quantification of expressions of Tf, FTH1 and GPX4 in the hearts of rats (*n* = 6 per group).

To identify specific microbiota associated with age-related HF, we conducted a comprehensive reanalysis of publicly available 16S rRNA sequencing data,^27^ focusing on 250 young and 98 elderly individuals. The principal coordinates analysis

(PCoA) of Unweighted UniFrac distances (β-diversity) among younger and older individuals showed that the composition of gut microbiota were significantly separated by aging, although no notable difference was observed on microbial α-diversity (Figure 2(a–b)). Notably, the abundance of *F. prausnitzii*, an important probiotic, was significantly lower in the elderly compared to their younger counterparts (Figure 2(c)). To measure the abundance of *F. prausnitzii*, we collected fecal samples from 40 healthy controls and 40 elderly patients with HF for PCR testing (Figure 2(d)). The results revealed a significant reduction in the abundance of *F. prausnitzii* in elderly patients with HF compared to healthy elderly individuals (Figure 2(e)). ROC curve analysis demonstrated that *F. prausnitzii* abundance could serve as a potential predictor for HF in elderly individuals, with an AUC value of 0.703 (Figure 2(f)). Additionally, the abundance of *F. prausnitzii* was positively correlated with left ventricular EF in patients (Figure 2(g)). These findings suggest that *F. prausnitzii* abundance was associated with HF.

The data are given as mean ± SEM and compared by Student’s t test or One way ANOVA.

*F. prausnitzii*, recognized for its anti-inflammatory properties and crucial role in maintaining intestinal homeostasis,^28,29^ has been linked to various diseases, including cardiovascular disorders.^30–32^ We tested its therapeutic potential in D-galactose-induced accelerated aging models. Subsequently, aging rats were randomly assigned to receive either a control solvent or *F. prausnitzii* supplementation (Figure 2(h)). The successful colonization of *F. prausnitzii* in the intestinal tract of supplemented rats was validated by PCR (Figure S5A). Compared with the control rats, aging rats exhibited impaired cardiac function and elevated plasma NT-proBNP levels, both of which were reversed by *F. prausnitzii* supplementation (Figure 2(i–k)). Furthermore, *F. prausnitzii* alleviated ferroptosis in aging rats, as showed by increased GSH levels, improved cardiac structural organization and restored mitochondrial integrity (Figure 2(l–m)). Additionally, *F. prausnitzii* upregulated cardiac expression of GPX4 and FTH1, while suppressing Tf expression (Figure 2(n)). To further substantiate that *F. prausnitzii* ameliorates cardiac dysfunction induced by age-related microbiota dysbiosis, we conducted mono-colonization experiments with *F. prausnitzii* in an aged microbiota transplantation model (Figure S5B). Notably, *F. prausnitzii* supplementation significantly increased the abundance of *F. prausnitzii*, and restored cardiac function in these rats (Figure S5C-F). These findings suggest that *F. prausnitzii* protects against age-related HF.

### F. prausnitzii-*derived butyrate mediates cardioprotection through ferroptosis regulation*

**Figure 3.**
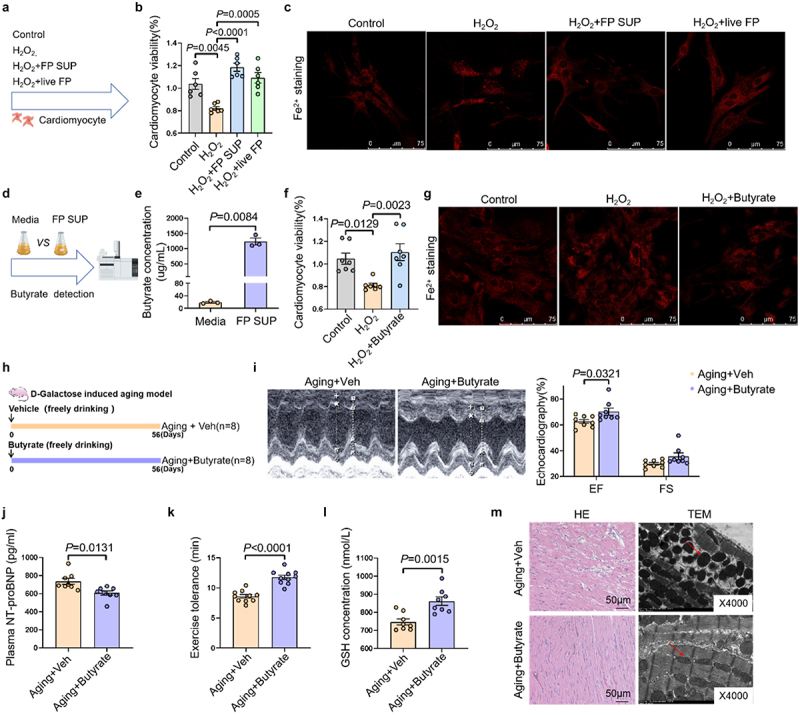
*F. prausnitzii-*derived butyrate alleviates ferroptosis and prevents age-related heart failure. (a) Cellular experiment schematic: H_2_O_2_ was used to establish a senescent cell model, and subsequently exposed to live *F. prausnitzii*, inactivated *F. prausnitzii*, and *F. prausnitzii* supernatant. (b) The cell viability of cardiomyocytes (*n* = 6 per group). (c) Representative images of FerroOrange detection in cardiomyocytes. (d) Butyrate detection by mass spectrometry: media (control) and *F. prausnitzii* supernatant (FP SUP) were collected for analysis to measure butyrate levels. (e) The concentration of butyrate in the media and *F. prausnitzii* supernatant (*n* = 7 per group). (f) The cell viability of cardiomyocytes in control group, H_2_O_2_ group, and H_2_O_2_ + butyrate group (*n* = 7 per group). (g) Representative image of FerroOrange detection in cardiomyocytes. (h) Schematic illustration of the experimental design. We established an aging rat model with D-galactose (150 mg/kg/day) via subcutaneous injection. The rats were randomly divided into two groups, received water containing butyrate sodium (1% w/w) for 8 weeks (Aging+butyrate group), another group received ordinary water (Aging+veh group). (i) Representative images of M-mode echocardiograms of hearts, and the statistical data of cardiac ejection fraction (EF) and cardiac fraction shortening (FS) of rats (*n* = 8 per group). (j) The plasma levels of NT-proBNP in rats (*n* = 8 per group). (k) The exercise tolerance in rats (*n* = 8 per group). (l) The levels of GSH in rats (*n* = 8 per group). (m) Representative images of HE staining, and transmission electron microscopy images of mitochondria in the heart of rats.

To dissect *F. prausnitzii’s* cardioprotective mechanism, we established a senescent cardiomyocyte model using hydrogen peroxide (H_2_O_2_). H_2_O_2_ pretreated cardiomyocytes were subsequently co-cultured with live *F. prausnitzii*, inactivated *F. prausnitzii*, or *F. prausnitzii* supernatant (Figure 3(a)). In H_2_O_2_-induced senescent cardiomyocyte, we observed a significant decline in cellular viability, accompanied by increased iron accumulation, both of which were effectively counteracted by live *F. prausnitzii* and its supernatant (Figure 3(b–c)). Notably, both live *F. prausnitzii* and its supernatant, which is devoid of bacteria, effectively mitigated iron accumulation, reduced Tf expression, and restored FTH1 and GPX4 levels caused by H_2_O_2_ exposure (Figure S6), indicating a direct effect of *F. prausnitizii*-derived metabolites on modulating ferroptosis. *F. prausnitzii* is known as a prominent contributor to butyrate generation. Consistent with this, GC-MS/MS analysis revealed a significant increase in butyrate levels in the *F. prausnitzii* supernatant (Figure 3(d–e)). To delineate the role of butyrate in ferroptosis, H_2_O_2_-pretreated cardiomyocytes were subjected to either a control solution or butyrate treatment. Butyrate effectively preserved cardiomyocyte viability (Figure 3(f)), reduced iron accumulation (Figure 3(g)), and upregulated GPX4 and FTH1, and suppressed Tf expression (Figure S7), further supporting its role in ferroptosis inhibition.

The data are given as mean ± SEM and compared by Student’s t test or One way ANOVA.

To further substantiate the potential of butyrate in age-related HF, we supplemented D-galactose-induced aging rats with butyrate in their drinking water (Figure 3(h)). Remarkably, butyrate treatment significantly rescued cardiac function, as evidenced by elevated EF, decreaed NT-proBNP, and enhanced exercise tolerance and GSH levels (Figure 3(i–l)). Furthermore, butyrate treatment restored cardiac structural integrity and mitochondrial morphology (Figure 3(m)).

### *The cardioprotective effects of* F. prausnitzii *are* *butyrate-dependent*

**Figure 4.**
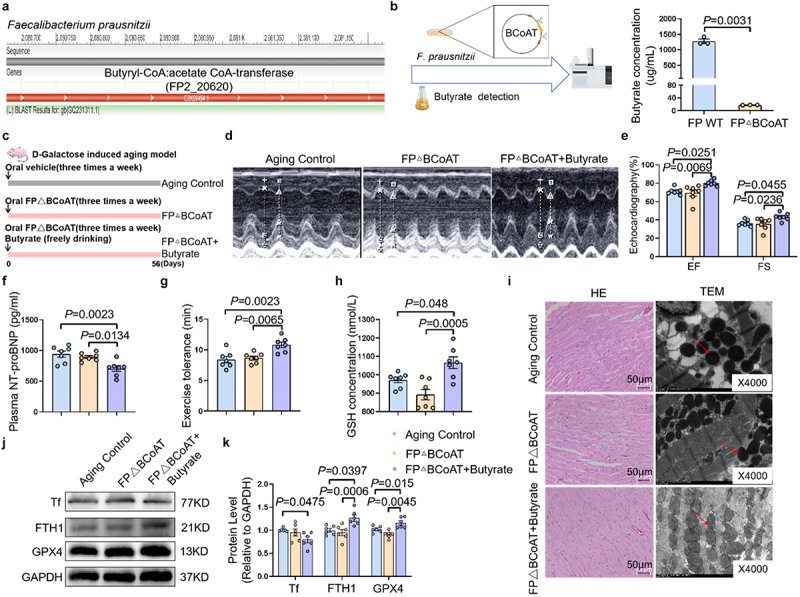
*F.Prausnitzii* prevents age-related heart failure through butyrate. (a) The genome of *F.Prausnitzii* L2–6 contained FP2_20620 encoding butyryl-CoA:acetate CoA-transferase. (b) Quantification of the concentration of butyrate in supernatant of *F.P* and *F.P*ΔBCoAT assessed by mass spectrometry (*n* = 4 per group). (c) Schematic illustration of the experimental design. We established an aging rat model with D-galactose (150 mg/kg/day) via subcutaneous injection. The rats were randomly divided into three groups, one group received *F.P*ΔBCoAT gavage for 8 weeks (*F.P*ΔBCoAT group), another group received *F.P*ΔBCoAT gavage combined with water containing butyrate sodium (1% w/w) for 8 weeks (*F.P*ΔBCoAT+Butyrate group), the aging control group received ordinary water. (d) Representative images of M-mode echocardiograms of hearts in rats. (e) The statistical data of cardiac ejection fraction (EF) and cardiac fraction shortening (FS) of rats (*n* = 7 per group). (f) The plasma levels of NT-proBNP in rats (*n* = 7 per group). (g) The exercise tolerance in rats (*n* = 7 per group). (h) The levels of GSH in rats (*n* = 7 per group). (i) Representative images of HE staining, and representative transmission electron microscopy images of mitochondria in the left ventricle of hearts in rats. The red arrow points to the mitochondria. (j) Representative bands showing the expressions of tf, FTH1 and GPX4 in the hearts of rats. (k) Quantification of the expressions of tf, FTH1 and GPX4 in the hearts of rats (*n* = 6 per group).

To determine the butyrate dependency of *F. prausnitzii’s* cardioprotection, we constructed a BCoAT-deficient mutant of the *F. prausnitzii* strain (*F.P*ΔBCoAT) using homologous recombination (Figure 4(a)). Metabolomic profiling by GC-MS/MS validated severe impairment of butyrate biosynthesis in the mutant (Figure 4(b)). In D-galactose-induced accelerated aging models, rats were administered vehicle control, *F.P*ΔBCoAT monotherapy, or *F.P*ΔBCoAT with exogenous butyrate supplementation (Figure 4(c)).

The data are given as mean ± SEM and compared by one way ANOVA.

Strikingly, *F.P*ΔBCoAT monotherapy failed to improve cardiac function, while concurrent butyrate administration completely restored cardiac performance. This phenotypic rescue paralleled reduced circulating NT-proBNP levels, improved exercise capacity, and normalized GSH content (Figure 4(d–h)). Cardiac structural remodeling remained unaltered by *F.P*ΔBCoAT treatment, whereas butyrate co-administration significantly mitigated age-related cardiac structural disarray (Figure 4(i)). Additionally, *F.P*ΔBCoAT monotherapy showed no modulatory effects on cardiac ferroptosis markers, in stark contrast to butyrate cotreatment which preserved mitochondrial integrity (Figure 4(i)), downregulated Tf expression, and upregulated ferroptosis suppressors FTH1 and GPX4 expression in aging rats (Figure 4(j–k)). Collectively, these findings demonstrate that *F. prausnitzii* attenuates age-related cardiac dysfunction via butyrate-dependent mechanisms, with specific involvement in ferroptosis regulation.

### Butyrate attenuates cardiomyocyte ferroptosis by regulating LCN2

**Figure 5.**
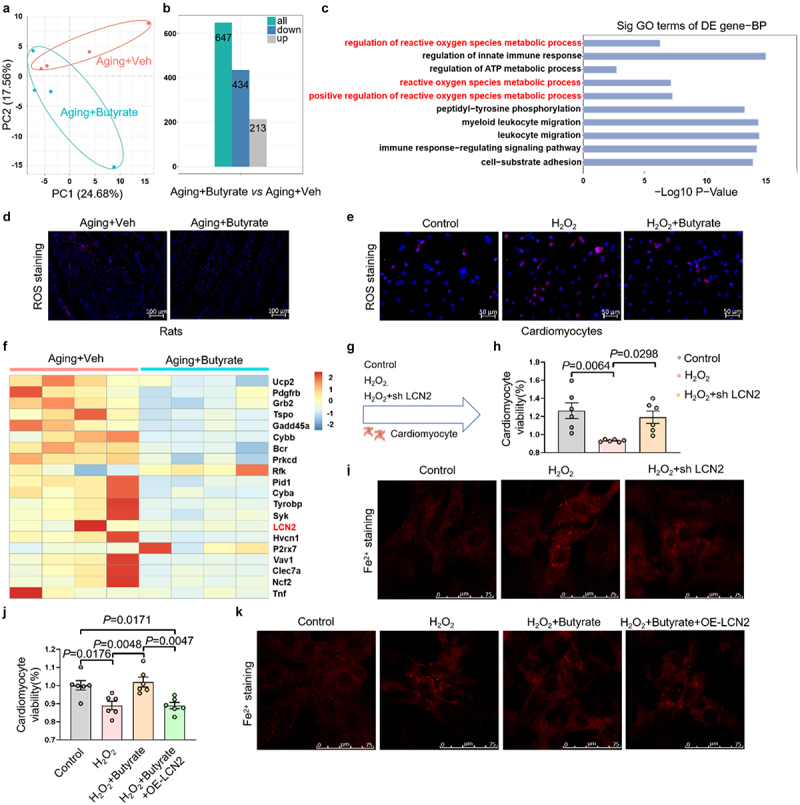
Butyrate inhibits ferroptosis by modulating LCN2 in cardiomyocytes. (a) Transcriptomics was performed to detect the gene expression changes. The volcano plot showed diferentially expressed genes between Aging+Veh group and Aging+Butyrate group. *n* = 4 biological replicates/group. (b) The statistical data shows the 213 up-regulated genes, 434 down-regulated genes between the two groups. (c) Gene ontology (GO) analysis for upregulated and downregulated genes in the RNA-seq data. (d) Representative ROS staining images and fluorescence intensity in the left ventricle of heart in rats. (e) Representative ROS staining images of cardiomyocytes. (f) Analysis of significantly differentially expressed genes in the oxidative stress pathway in the left ventricle of hearts of rats from Aging+Veh group and Aging+Butyrate group. (g) Cardiomyocytes were transfected with plasmids to silence LCN2. After 24 h, they were treated with H_2_O_2_ for 6 h. Following a change of culture media, the cells were further incubated for 42 h before conducting assays for relevant markers. (h) The viability of cardiomyocytes (*n* = 6 per group). (i) Representative image of FerroOrange detection in cardiomyocytes. (j) Cardiomyocytes were first transfected with plasmids for LCN2 overexpression. After 24 h, they were treated with H_2_O_2_ for 6 h. Following a change of culture media, the cells in H_2_O_2_+butyrate group and H_2_O_2_+butyrate+OE-LCN2 group were treated with butyrate for 42 h before conducting assays for relevant markers. The viability of cardiomyocytes (*n* = 6 per group). (k) Representative image of FerroOrange detection in cardiomyocytes.

To elucidate the molecular basis of butyrate-mediated cardioprotection in aging, we conducted cardiac transcriptomic profiling in D-galactose-induced aging rats with or without butyrate intervention. Comparative analysis identified 647 differentially expressed genes (213 upregulated, 434 downregulated) post-butyrate treatment (Figure 5(a–b)). Pathway enrichment analysis revealed the reactive oxygen species (ROS) metabolic process as the most enriched pathway (Figure 5(c)), consistent with observed ROS attenuation in both ventricular tissues of butyrate-treated aging rats (Figure 5(d)) and H_2_O_2_-induced senescent cardiomyocytes (Figure 5(e)). These data indicate that butyrate mitigates ROS-driven ferroptosis.

The data are given as mean ± SEM and compared by Student’s t test or One way ANOVA.

Strikingly, lipocalin-2 (LCN2), a regulator of oxidative stress and iron metabolism,^33^ ranked among the most downregulated genes in the ROS metabolic pathway following butyrate treatment (Figure 5(f)). Previous studies have demonstrated that LCN2 can induce ferroptosis and contribute to various diseases.^34,35^ Intriguingly, we observed a significant elevation in the expression of LCN2 in both the aged rat hearts and H_2_O_2_-induced senescent cardiomyocytes (Figure S8A-B). To further explore the role of LCN2, we employed small interfering RNA to knock it down (Figure 5(g)). H_2_O_2_-induced senescent cardiomyocytes exhibited decreased cell viability, increased iron deposition and ROS accumulation, while silencing LCN2 abrogated these H_2_O_2_-induced detrimental effects in cardiomyocytes (Figure 5(h–i), and Figure S9A-B). Moreover, silencing LCN2 also rescued the expression of Tf, FTH1, and GPX4 (Figure S9C). To establish butyrate-LCN2- ferroptosis axis causality, we engineered a LCN2-overexpressing plasmid. Notably, butyrate treatment effectively restored cell viability, reduced H_2_O_2_-induced iron deposition (Figure 5(j–k)). Moreover, butyrate suppressed ROS levels, and rectified ferroptosis-related protein expression in cardiomyocytes (Figure S10A-C). Crucially, LCN2 overexpression significantly counteracted butyrate’s protective effects in senescent cardiomyocytes. Taken together, butyrate alleviates cardiomyocyte ferroptosis by downregulating LCN2 expression.

## Discussion

**Figure 6.**
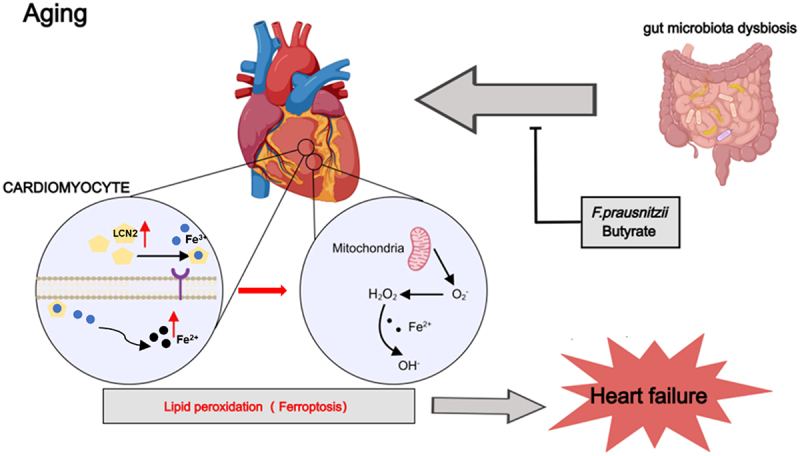
*Faecalibacterium prausnitzii* prevents age-related heart failure through inhibition of ferroptosis by butyrate. Aged microbiota dysbiosis heightens ferroptosis and increases susceptibility to HF in rats. Oral supplementation of *F. prausnitzii* mitigates ferroptosis and confers protection against age-related HF through butyrate by downregulating LCN2 in senescent cardiomyocyte.

This study uncovers a causal link between age-related gut dysbiosis and HF progression, identifying ferroptosis as a central pathological mechanism and proposing *F. prausnitzii-*butyrate supplementation as a promising therapeutic strategy. Integrated single-cell transcriptomics and functional validation reveal that aged-related microbiota dysbiosis drives cardiac dysfunction by promoting ferroptosis, while *F. prausnitzii* or butyrate administration rescues this phenotype through regulation of LCN2 modulation in cardiomyocytes (Figure 6). These findings advance our understanding of gut-heart crosstalk in aging while offering microbiota-targeted interventions for HF management.

HF is a common cardiovascular condition in the elderly, with its incidence rising significantly with age.^36^ It contributes to a large number of hospitalizations and mortality, especially in individuals aged 75 and older.^37,38^ However, the mechanism underlying age-related HF remains enigmatic. The gut microbiota undergoes significant transformations with aging, and this microbial dysbiosis is increasingly implicated in age-related diseases.^39–41^ Intriguingly, our research uncovered that aged microbiota transplantation induces a decline in cardiac function in young recipients, suggesting that gut ecology as a modifiable driver of age-related HF. This aligns with emerging paradigms where microbiota-targeted therapies reverse aging phenotypes in multiple organ systems,^7,42,43^ now extended to cardiovascular pathophysiology.

*F. prausnitzii* is a commensal bacterium abundant in the intestinal microbiota of healthy adults, known for its butyrate production and associated anti-inflammatory properties.^28,31^ We revealed a remarkable deletion of *F. prausnitzii* in elderly subjects and patients with HF, and its supplementation appeared to protect rats from age-related HF. This aligns with previous literature that reported the cardioprotective effects post-infarction conferred by butyrate-producing bacteria.^44^ *F. prausnitzii* exhibited beneficial effects in attenuating inflammatory bowel disease and chronic kidney disease via butyrate.^28,45^ Moreover, butyrate has been revealed to diminish atherosclerotic plaque sizes and protect ApoE^−/−^ mice from atherosclerosis.^46^ In the present study, we found that supplementation with *F. prausnitzii* or butyrate alleviated ferroptosis and prevented rats from age-related HF. Crucially, genetic disruption of butyrate synthesis (*F.PΔBCoAT*) abolishes therapeutic efficacy, resolving longstanding ambiguities about microbial metabolite specificity in cardiovascular contexts. Therefore, supplementation of *F. prausnitzii* or butyrate may be an effective therapy for age-related HF.

Ferroptosis, a lipid peroxidation-driven cell death process,^47^ has been mechanistically linked to age-related diseases, including Alzheimer’s and Parkinson’s disease.^48–50^ Furthermore, ferroptosis has been implicated in various cardiovascular diseases, such as atherosclerosis, drug-induced HF, myocardial ischemia-reperfusion injury, and cardiomyopathy.^51–53^ Our work establishes aged microbiota as a novel ferroptosis inducer and identifies butyrate as its microbial-derived suppressor, and highlights the important role of aged microbiota-induced ferroptosis in HF.

Lipocalin-2 (LCN2), a secretory protein within the lipocalin family consisting of 160–180 amino acids,^54^ has been increasingly implicated in various cardiovascular diseases.^55,56^ Elevated level of LCN2 is identified as an independent predictor of mortality in patients with acute coronary syndrome and as an early biomarker for cardiorenal syndrome in patients with acute HF.^57,58^ LCN2 influences ferroptosis through the regulation of iron accumulation and subsequent oxidative damage.^59^ Myeloid-specific knockout of LCN2 prevented ferroptosis and tissue wasting in lung cancer cachexia mice.^34^ Elevated LCN2 in retinal pigmented epithelial cells contributed to oxidative stress-induced ferroptosis processes in a mouse model of dry age-related macular degeneration.^60^ Our work identifies LCN2 as the critical butyrate-sensitive node. Elevated LCN2 in aged hearts and senescent cardiomyocytes aligns with its established role as a cardiovascular risk predictor. These findings highlight the pivotal role of LCN2-induced ferroptosis in age-related HF and shed light on the cardioprotective mechanism of butyrate.

## Conclusions

Our study provides groundbreaking insights into the crucial role of gut microbiota dysbiosis in the progression of age-related HF, highlighting the pivotal role of *F. prausnitzii* and its metabolite butyrate in mitigating ferroptosis-driven cardiac dysfunction. Our study suggest that *F. prausnitzii* as a potential microbial marker for HF underscores its translational relevance and paves the way for microbiota-based diagnostics and therapeutics. These findings not only deepen our understanding of the gut-heart axis but also highlight the potential of targeting gut microbiota and its metabolites to combat age-related cardiovascular diseases.

## Limitations

Our study has limitations. While our study focused on cardiomyocytes and ferroptosis, single-cell transcriptomics revealed significant changes in other cardiac cell types with aging, which were not further explored. Future investigations into the roles of these cell types in cardiac aging and HF are warranted to provide a more comprehensive understanding of the cellular landscape in aging hearts. Another limitation of this study is the exclusive use of male rats. This design was based on prior evidence suggesting female rodents exhibit resistance to iron overload-induced cardiomyopathy and the potential confounding influence of estrogen in age-related pathologies. However, whether such sex-specific protection extends to age-related heart failure remains unclear, warranting future investigations in female models.

## Supplementary Material

Supplemental Material

## Data Availability

Data will be made available on request.
