# Supplementary material for: Faecalibacterium prausnitzii prevents age-related heart failure by suppressing ferroptosis in cardiomyocytes through butyrate-mediated LCN2 regulation
Source: Gut Microbes. 2025 May 13;17(1):2505119. doi: 10.1080/19490976.2025.2505119 (PMC12080280; doi:10.1080/19490976.2025.2505119)
Supplement: Supplemental Material [file KGMI_A_2505119_SM8129.zip › Supplementary_Materials_of_Original_Data.docx]

Tf

GAPDH

130KD

95KD

72KD

55KD

40KD

33KD

25KD

15KD

10KD

Tf

GAPDH

130KD

95KD

72KD

55KD

40KD

33KD

25KD

15KD

10KD

Tf GAPDH


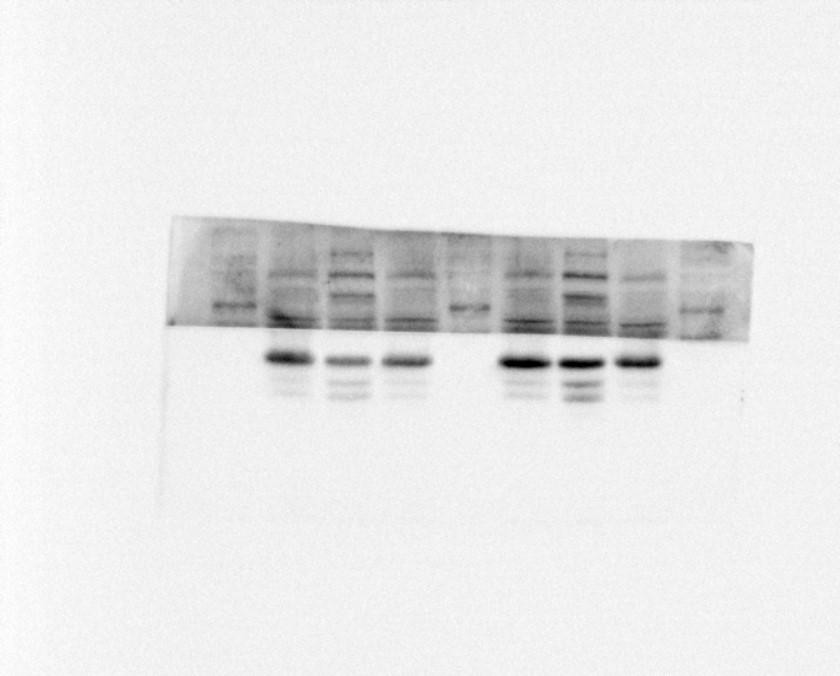

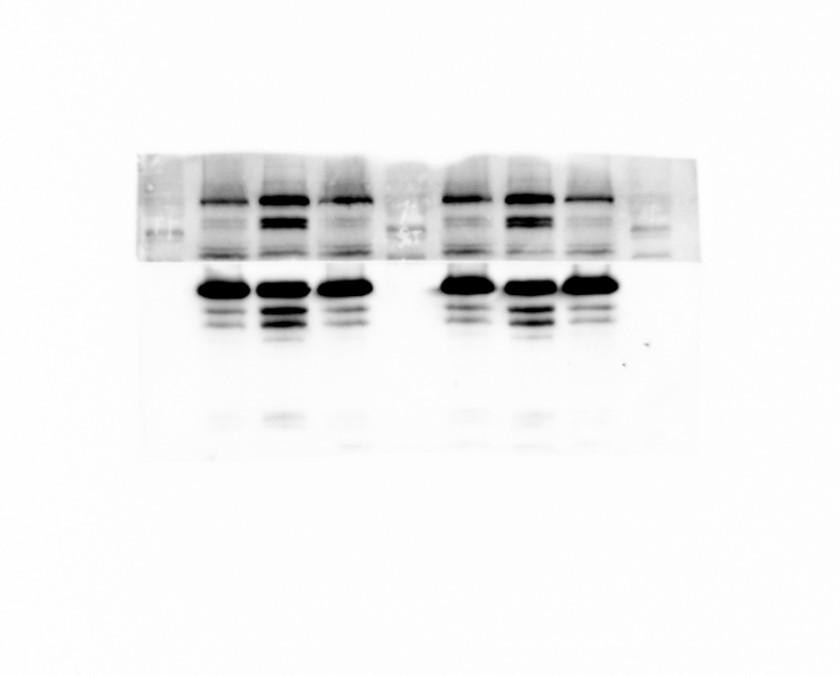

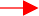

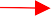

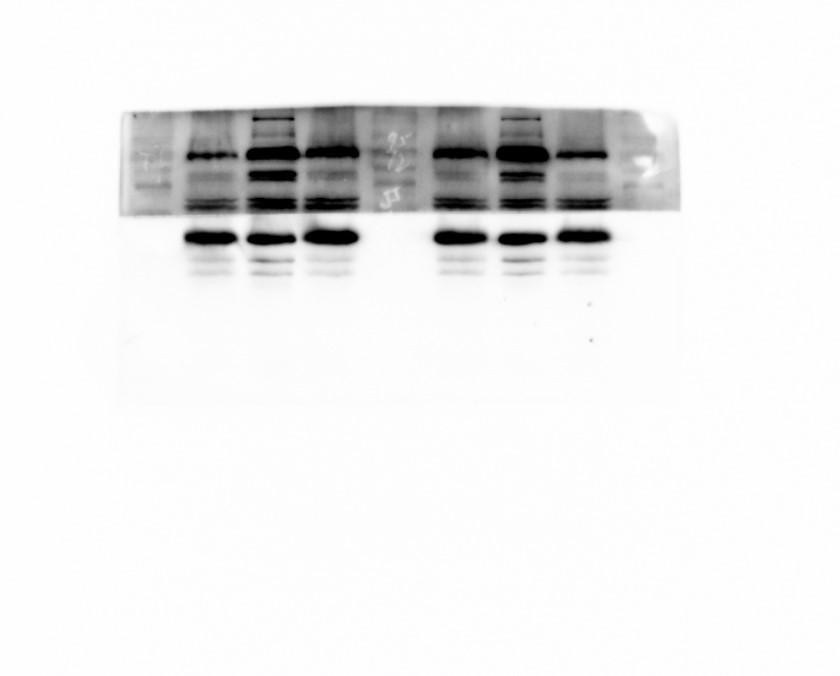

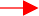

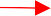

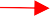


95KD

72KD

55KD

40KD

33KD

25KD

15KD

10KD


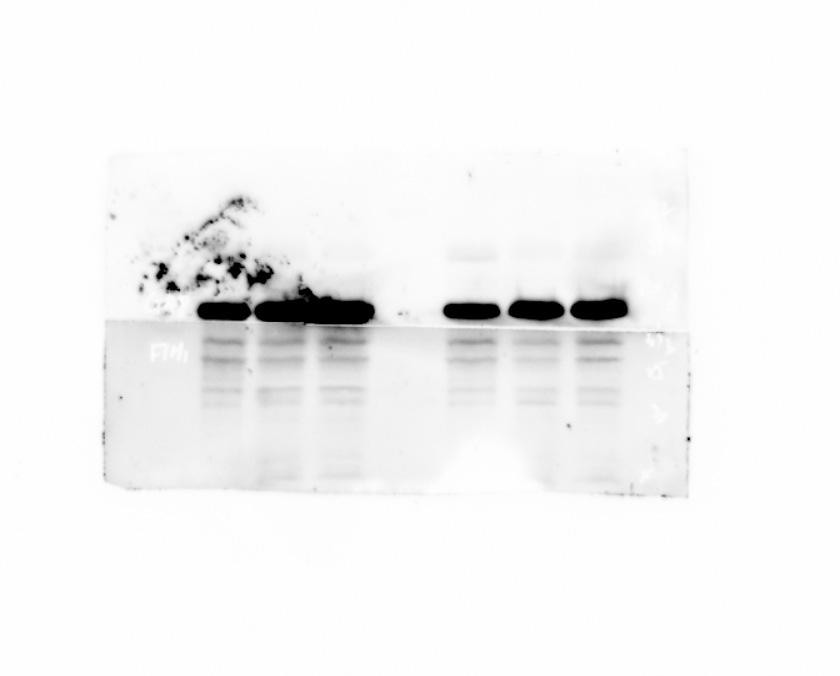

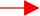

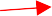


130KD

95KD

72KD

55KD

GAPDH

FTH1

40KD

33KD

25KD

15KD


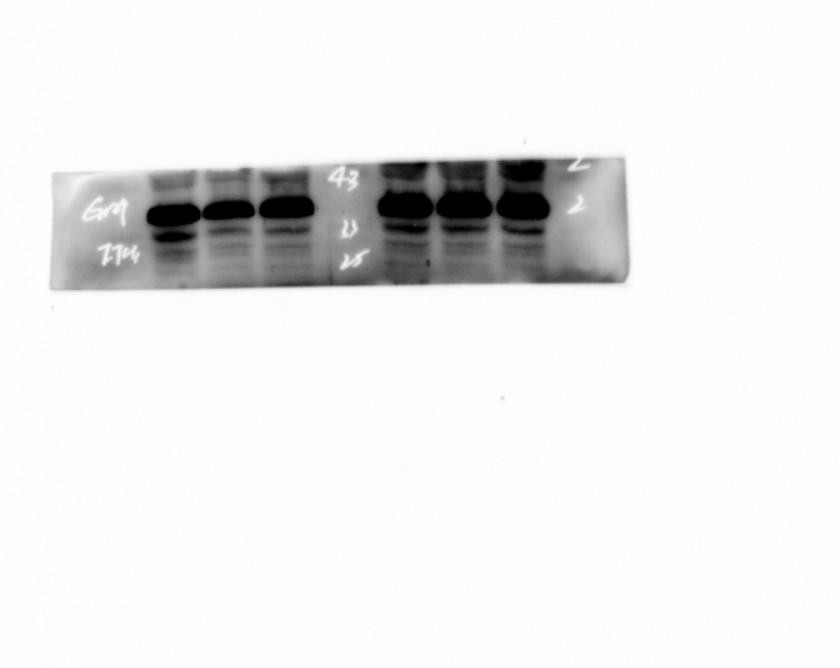

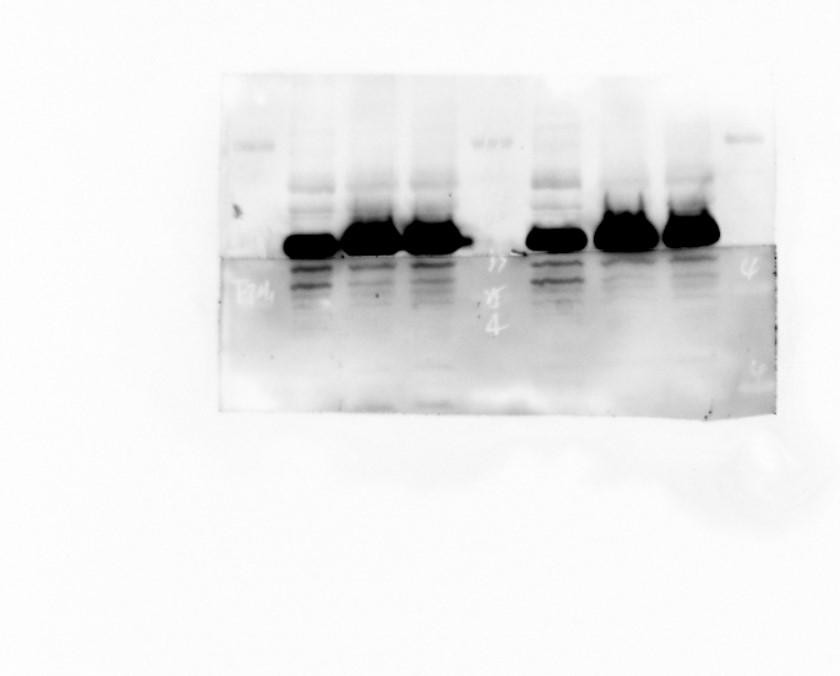

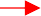

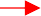

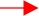


130KD

95KD

72KD

55KD

GAPDH

FTH1

40KD

33KD

25KD

15KD

130KD

95KD

72KD

55KD

40KD

GAPDH

FTH1

33KD

25KD

15KD


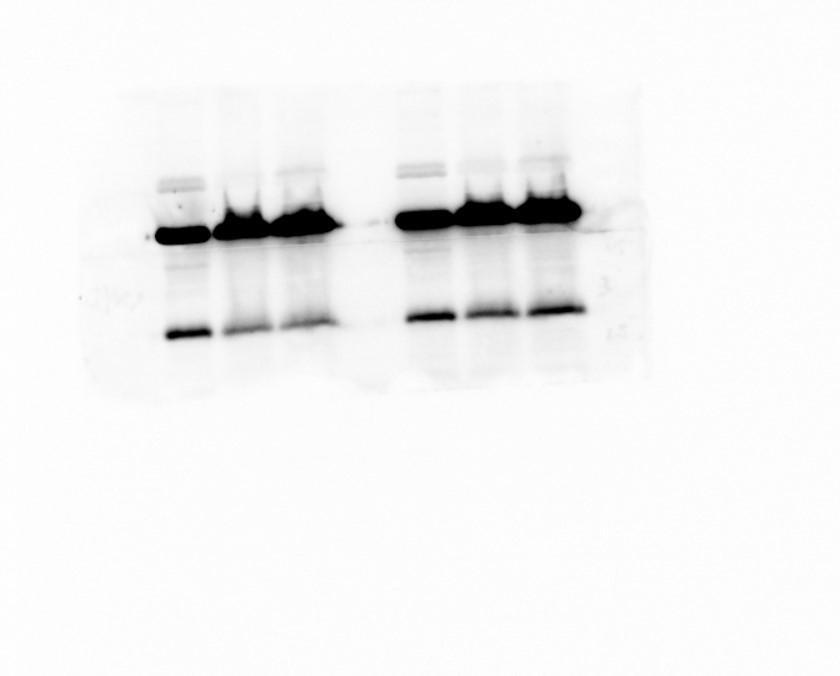

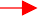

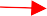


GAPDH

130KD

95KD

72KD

55KD

40KD

33KD

25KD

GPX4

15KD


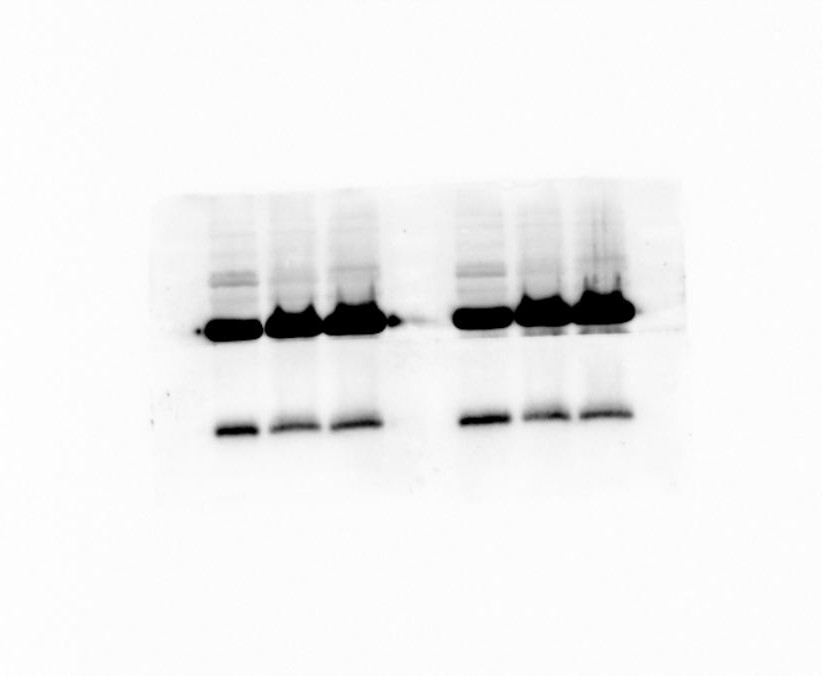

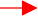

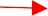

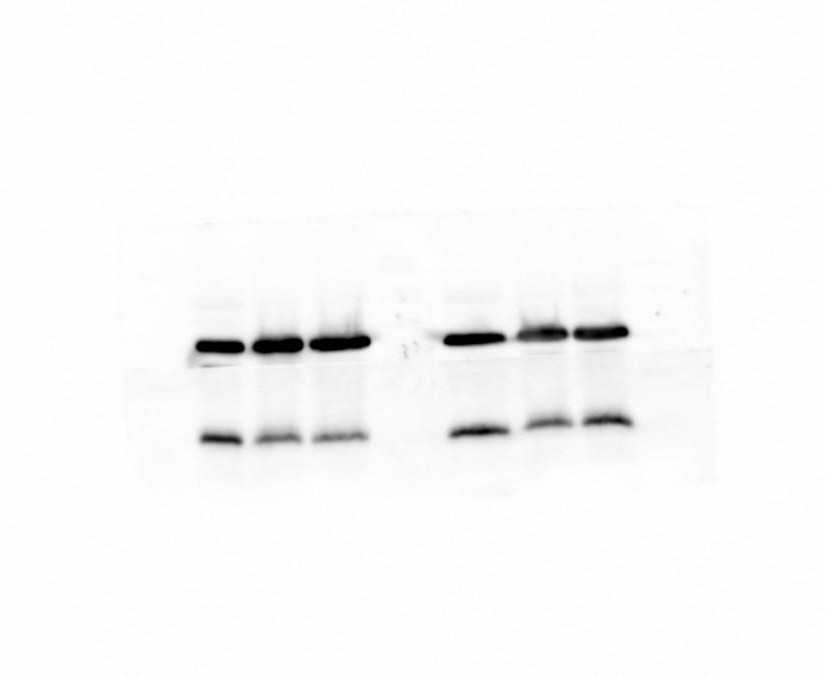


GAPDH

130KD

95KD

72KD

55KD

40KD

33KD

25KD

GPX4

15KD

GAPDH

130KD

95KD

72KD

55KD

40KD

33KD

25KD

GPX4

15KD

Tf

GAPDH

130KD

95KD

72KD

55KD

40KD

33KD

25KD

15KD

10KD

Tf

GAPDH

130KD

95KD

72KD

55KD

40KD

33KD

25KD

15KD

10KD

Tf

GAPDH

130KD

95KD

72KD

55KD

40KD

33KD

25KD

15KD

10KD


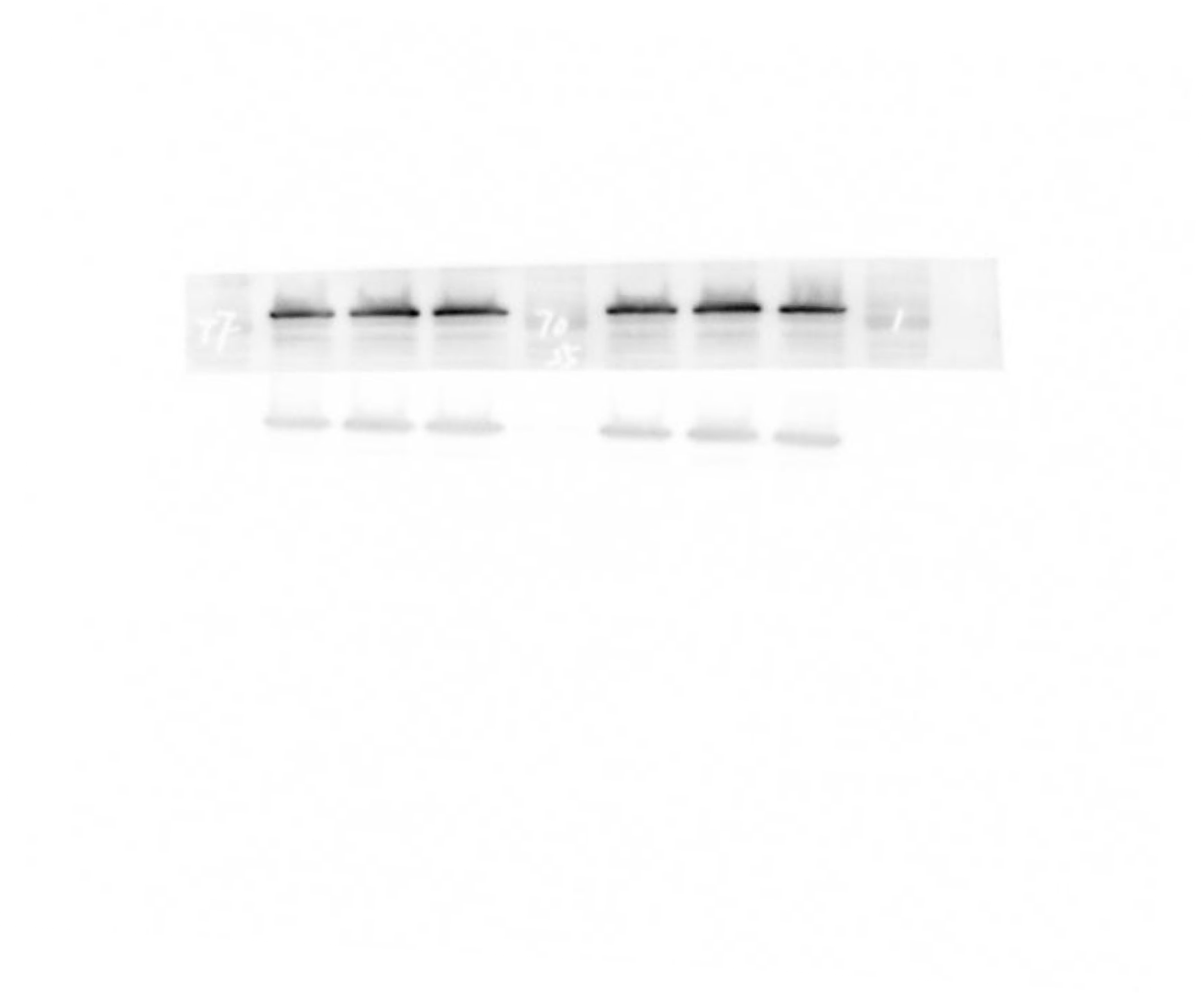

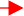

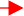

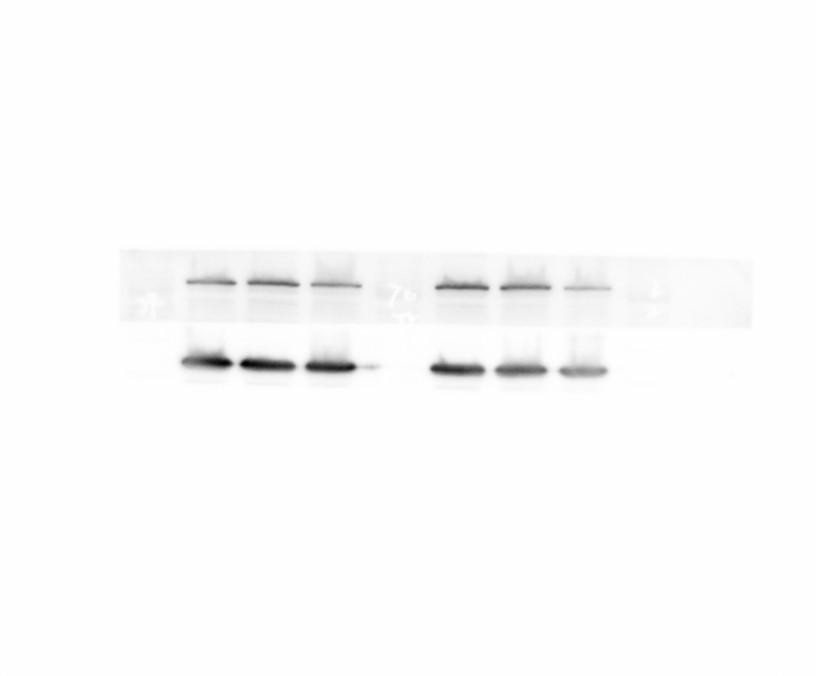

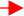

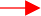

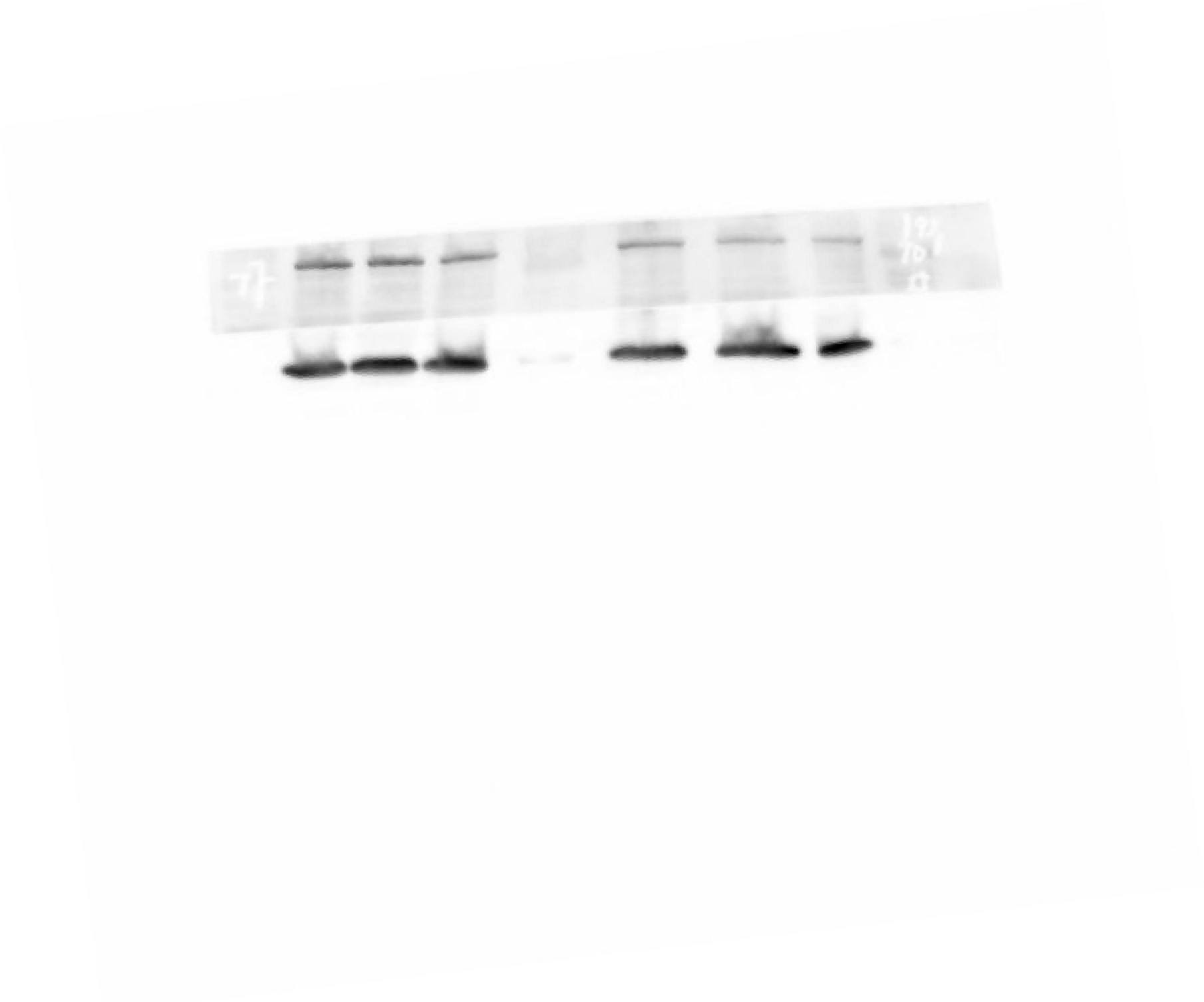

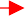

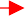

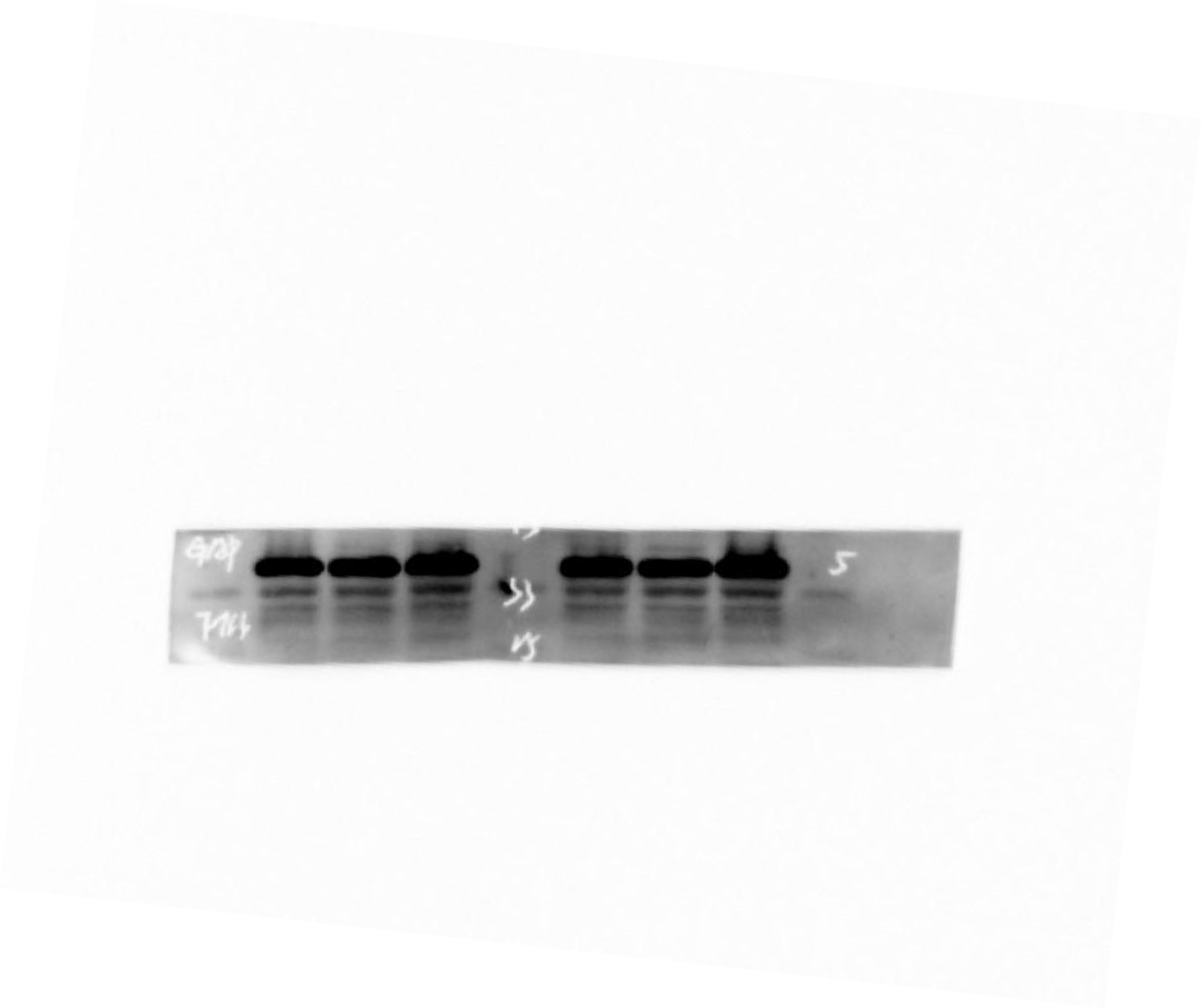

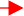

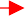

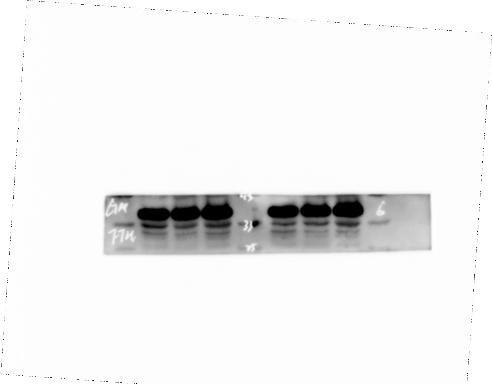

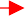

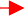


**Figure4 J——FTH1**

130KD

95KD

72KD

55KD

40KD

GAPDH

FTH1

33KD

25KD

15KD

130KD

95KD

72KD

55KD

40KD

GAPDH

FTH1

33KD

25KD

15KD


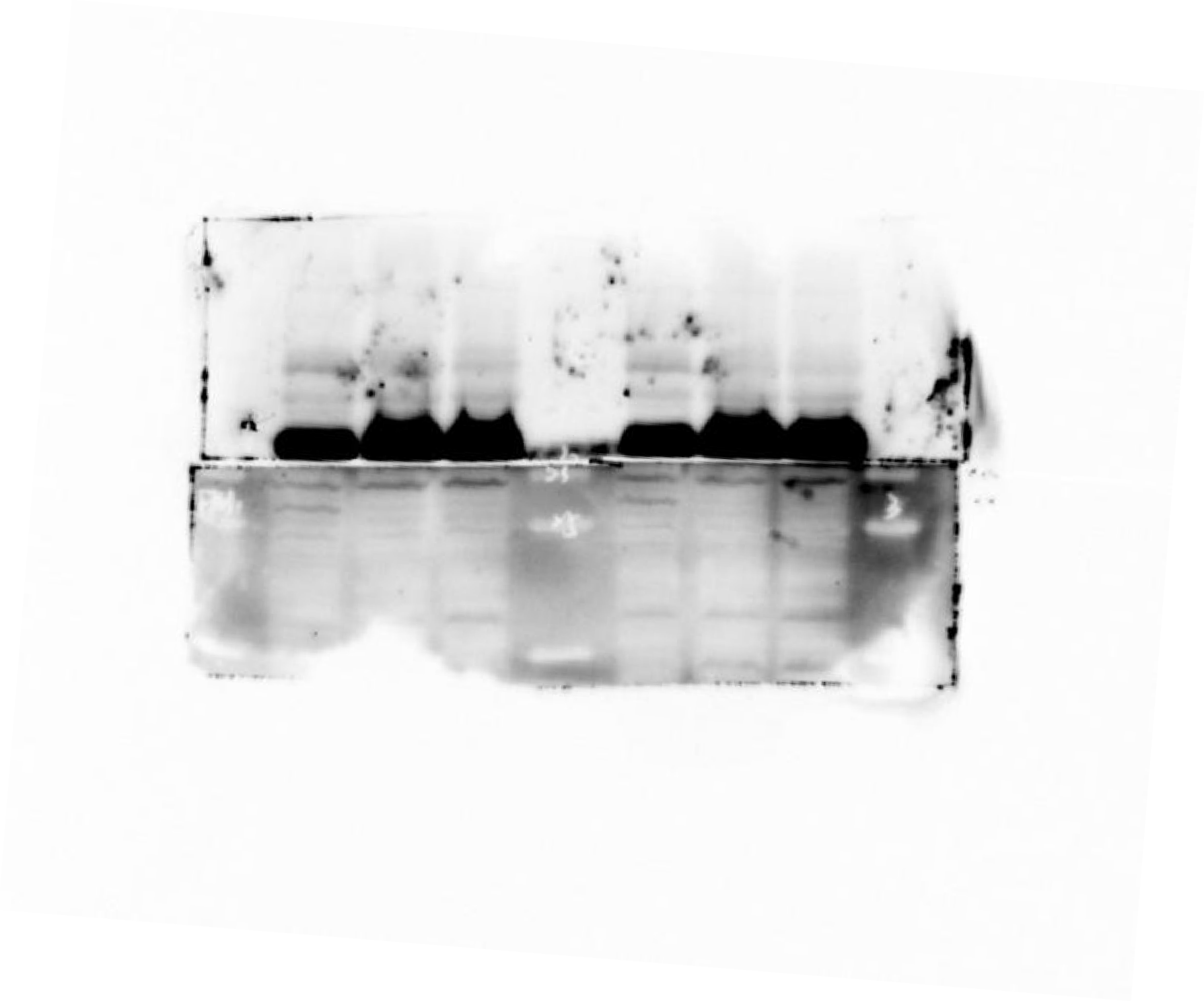

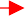

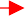


130KD

95KD

72KD

55KD

40KD

GAPDH

FTH1

33KD

25KD

15KD

**Figure4 J——GPX4**


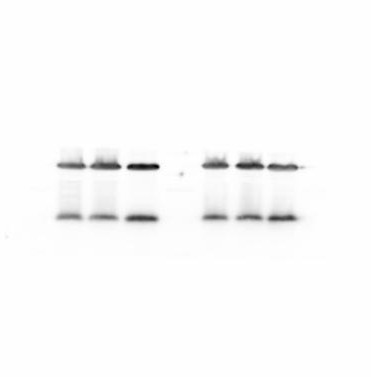

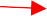

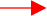


130KD

95KD

72KD

55KD

40KD

GAPDH

33KD

25KD

GPX4

15KD


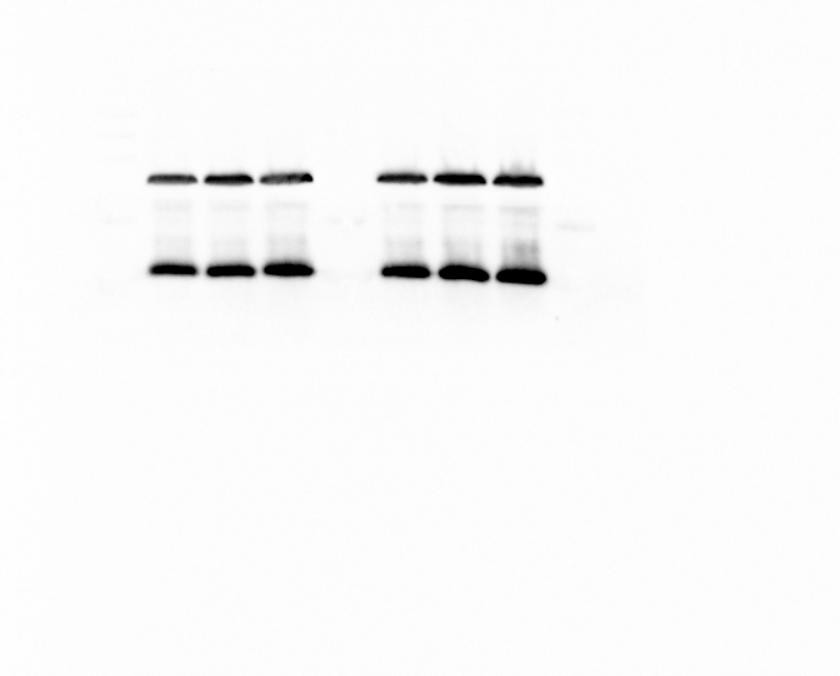

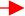

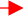

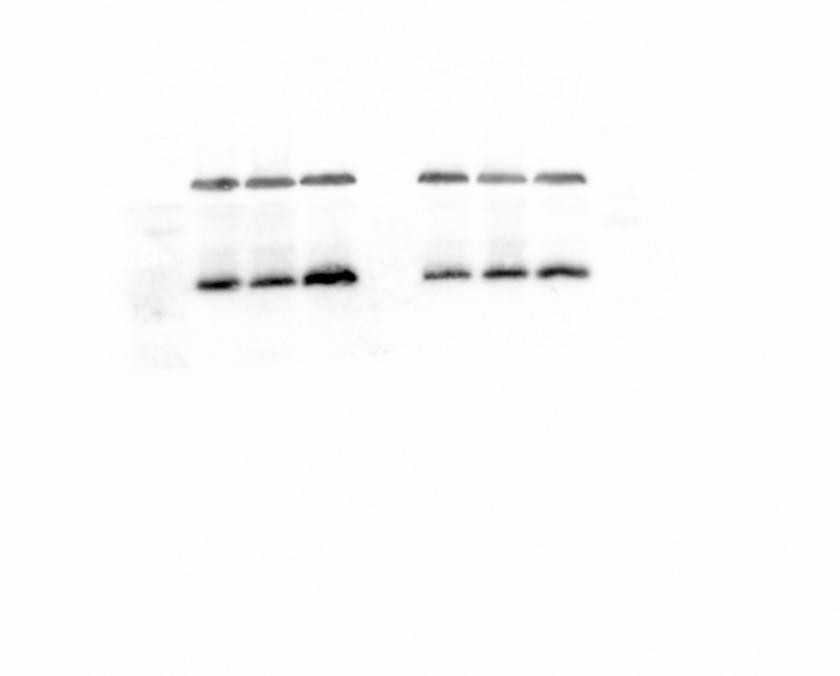

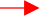


GAPDH

130KD

95KD

72KD

55KD

40KD

GPX4

33KD

25KD

15KD

130KD

95KD

72KD

55KD

40KD

GAPDH

33KD

25KD

GPX4

15KD


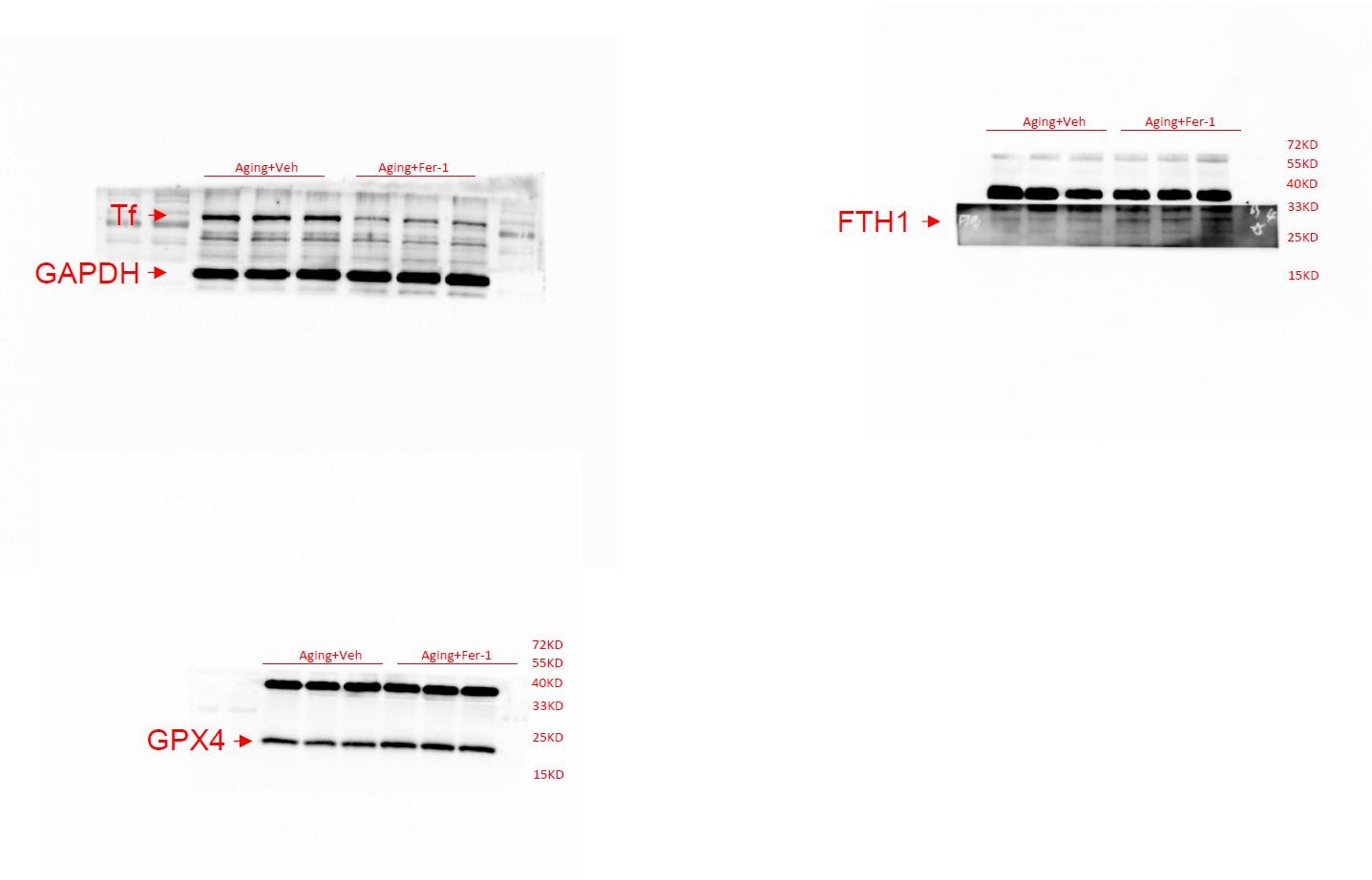


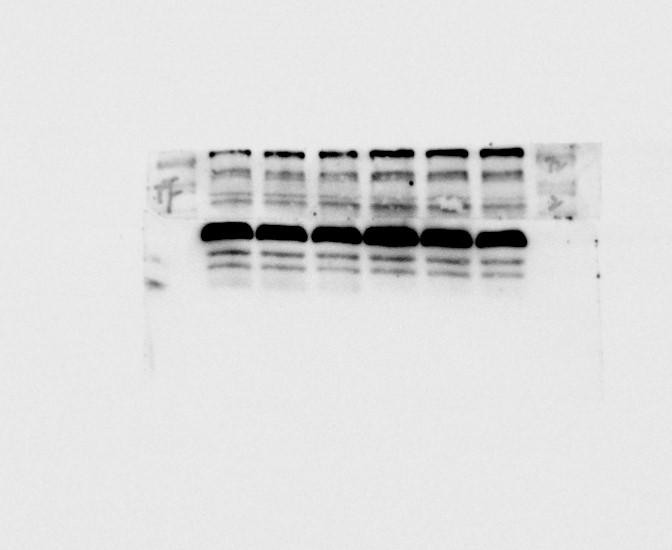

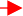

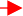

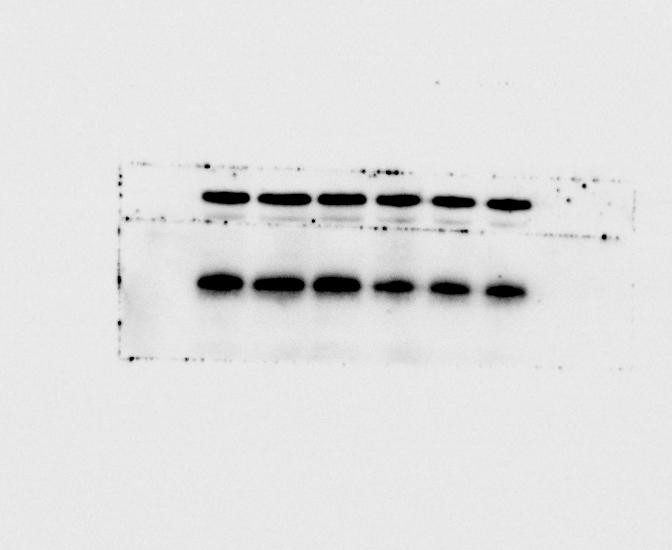

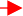

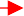


Young-FMT Aging-FMT

Tf

Young-FMT

Aging-FMT

72KD

55KD

GAPDH

40KD

40KD

GAPDH

33KD

25KD

33KD

25KD

GPX4

15KD

15KD


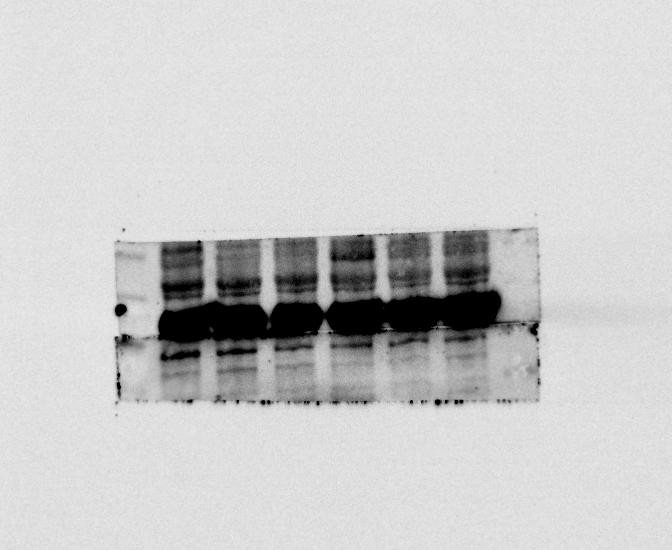

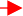

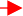


Young-FMT Aging-FMT

55KD

40KD

33KD

GAPDH

FTH1

25KD


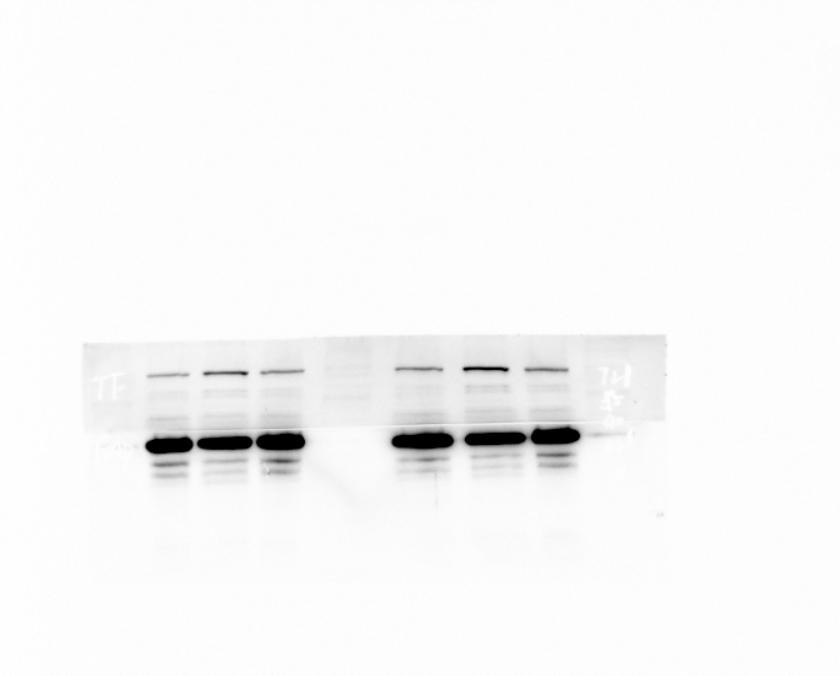

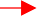

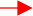

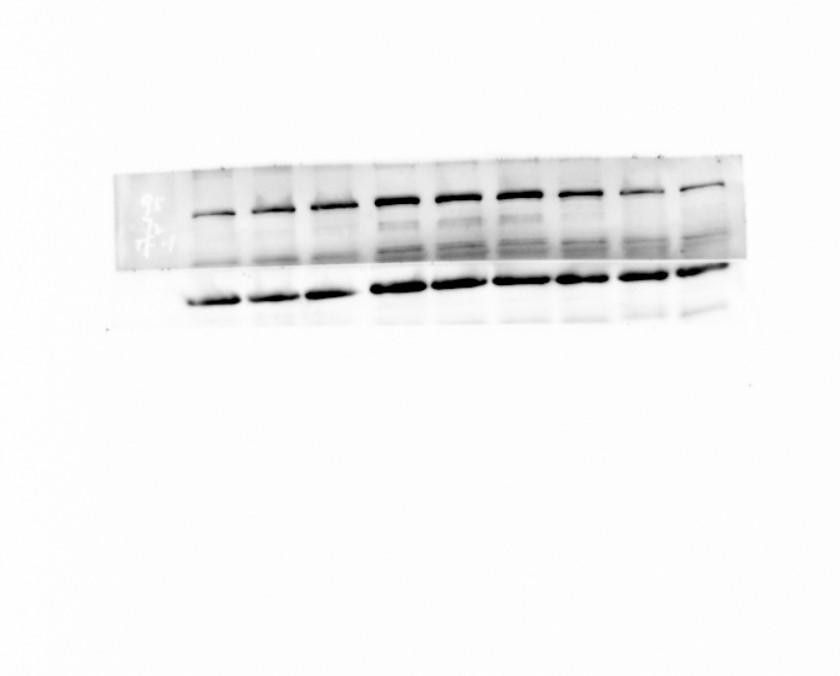

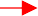

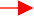


**FigureS4 ——Tf**

Tf

GAPDH

72KD

55KD

40KD

33KD

25KD

15KD

**Healthy Control**

Tf

GAPDH

72KD

55KD

40KD

33KD

25KD

15KD


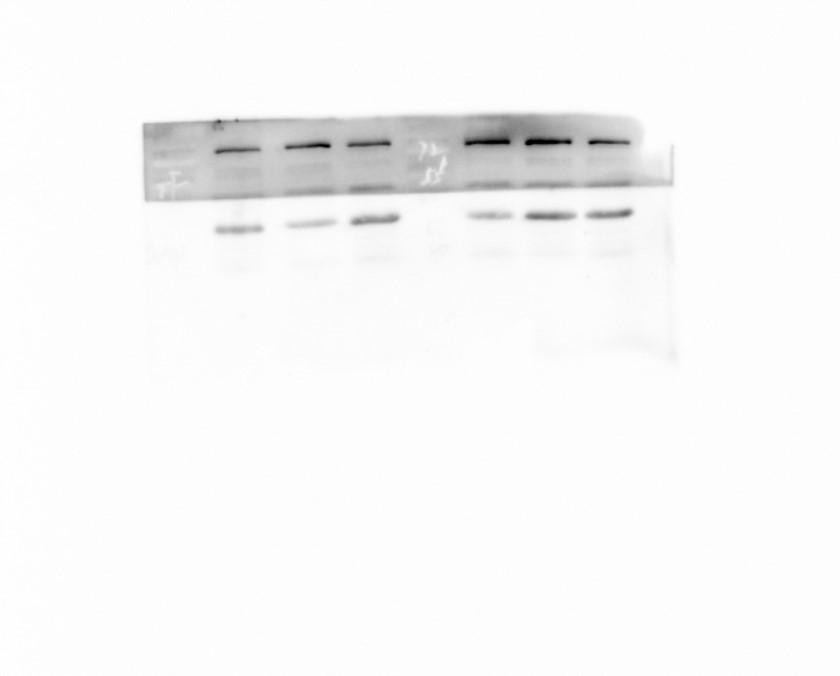

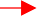

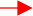


Tf

GAPDH

72KD

55KD

40KD

33KD

25KD

15KD

GAPDH FTH1

130KD

95KD

72KD

55KD

40KD

33KD

25KD

15KD

GAPDH FTH1

130KD

95KD


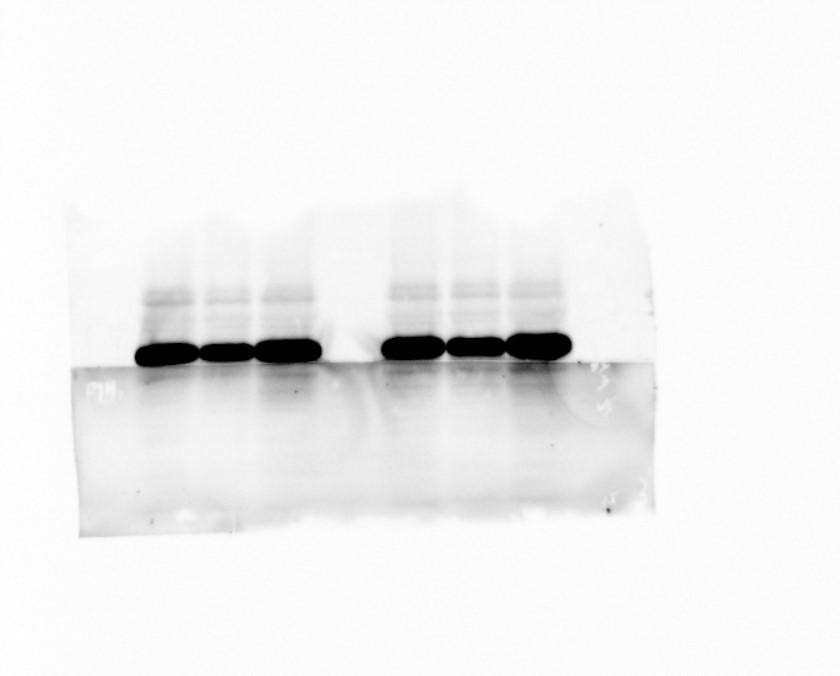

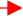

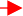


130KD

95KD

72KD

55KD

40KD

GAPDH

FTH1

33KD

25KD

15KD

72KD

55KD

40KD

33KD

25KD

15KD

130KD

95KD

72KD

55KD

40KD

GAPDH FTH1


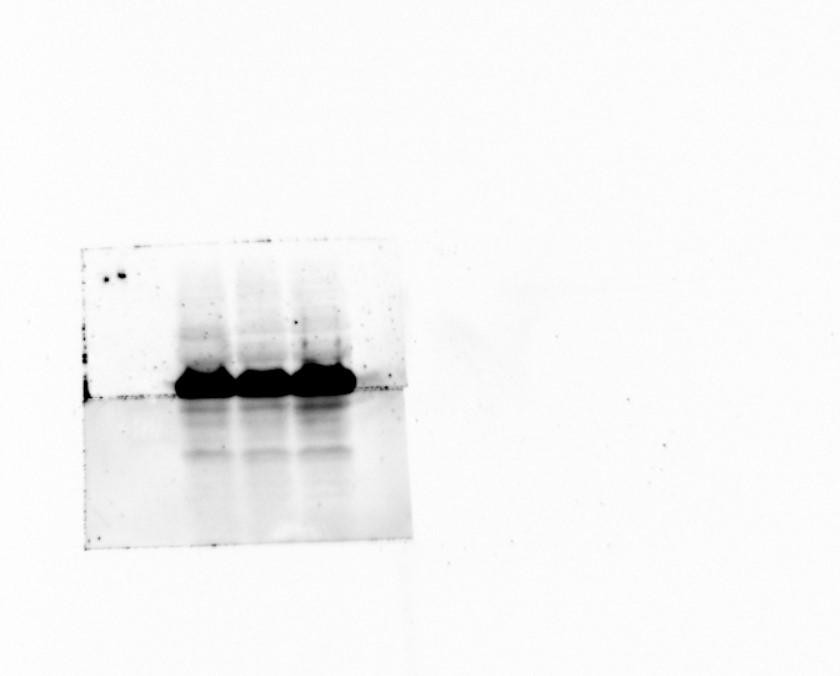

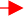

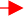

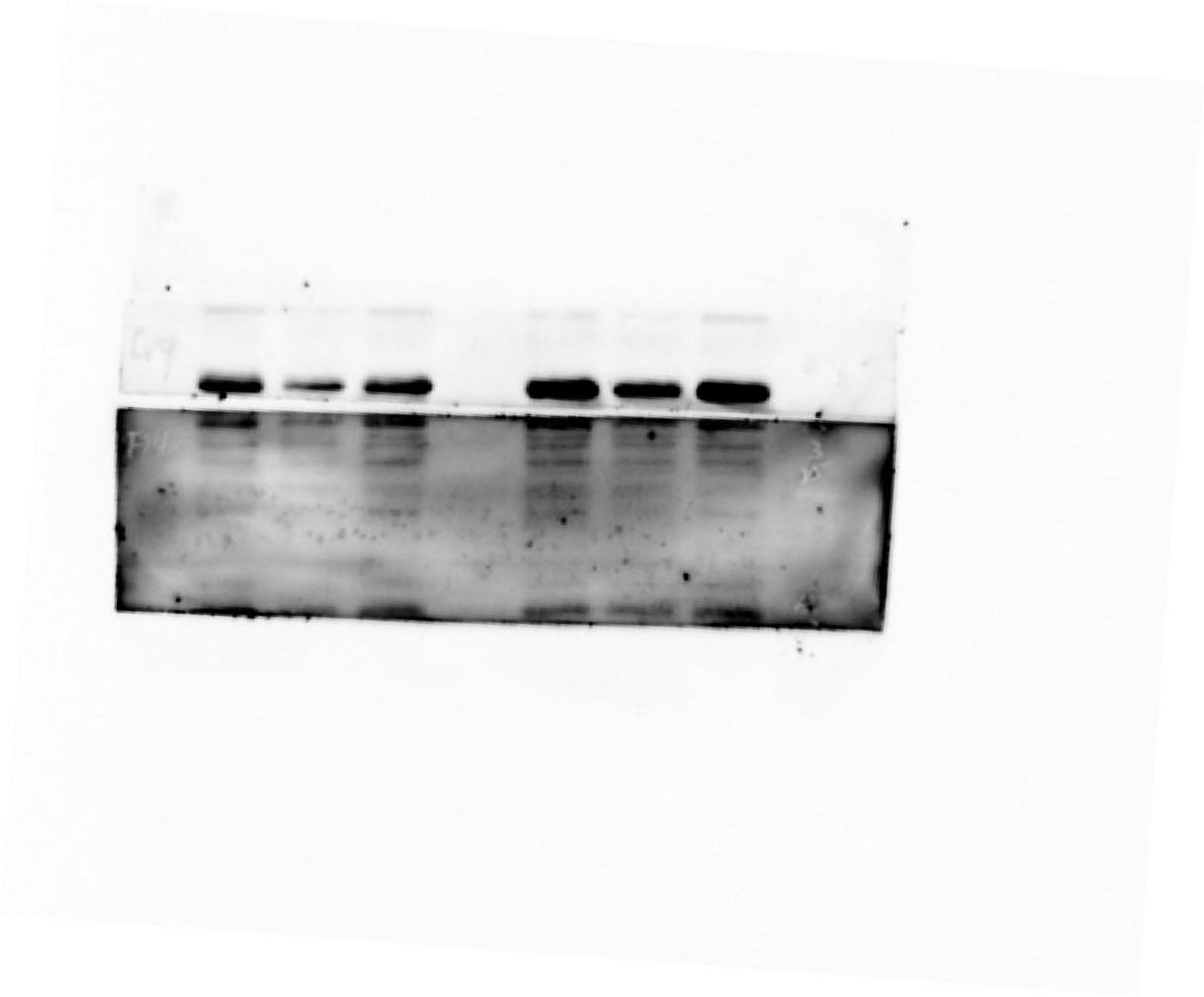

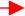

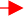

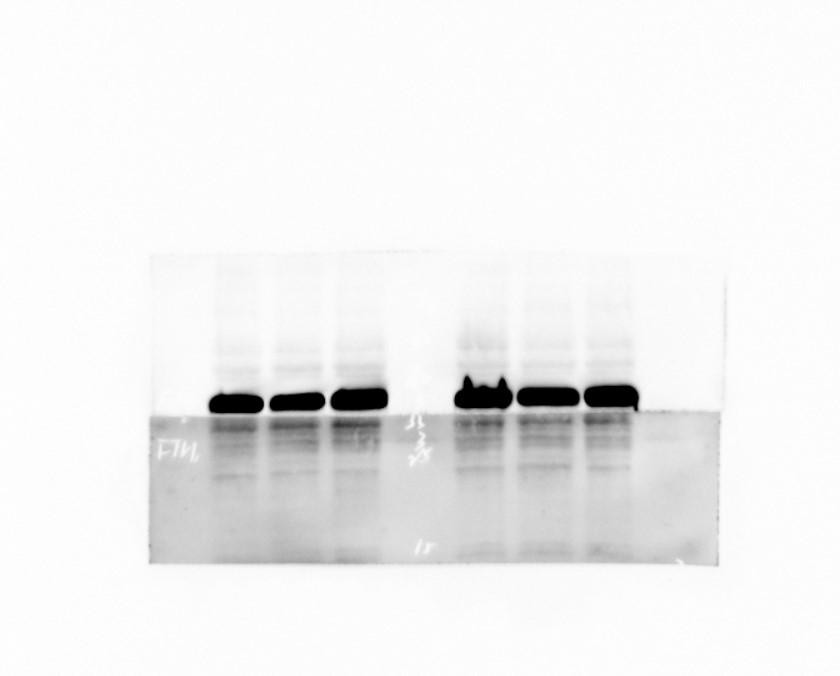

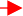

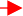


33KD

25KD

15KD


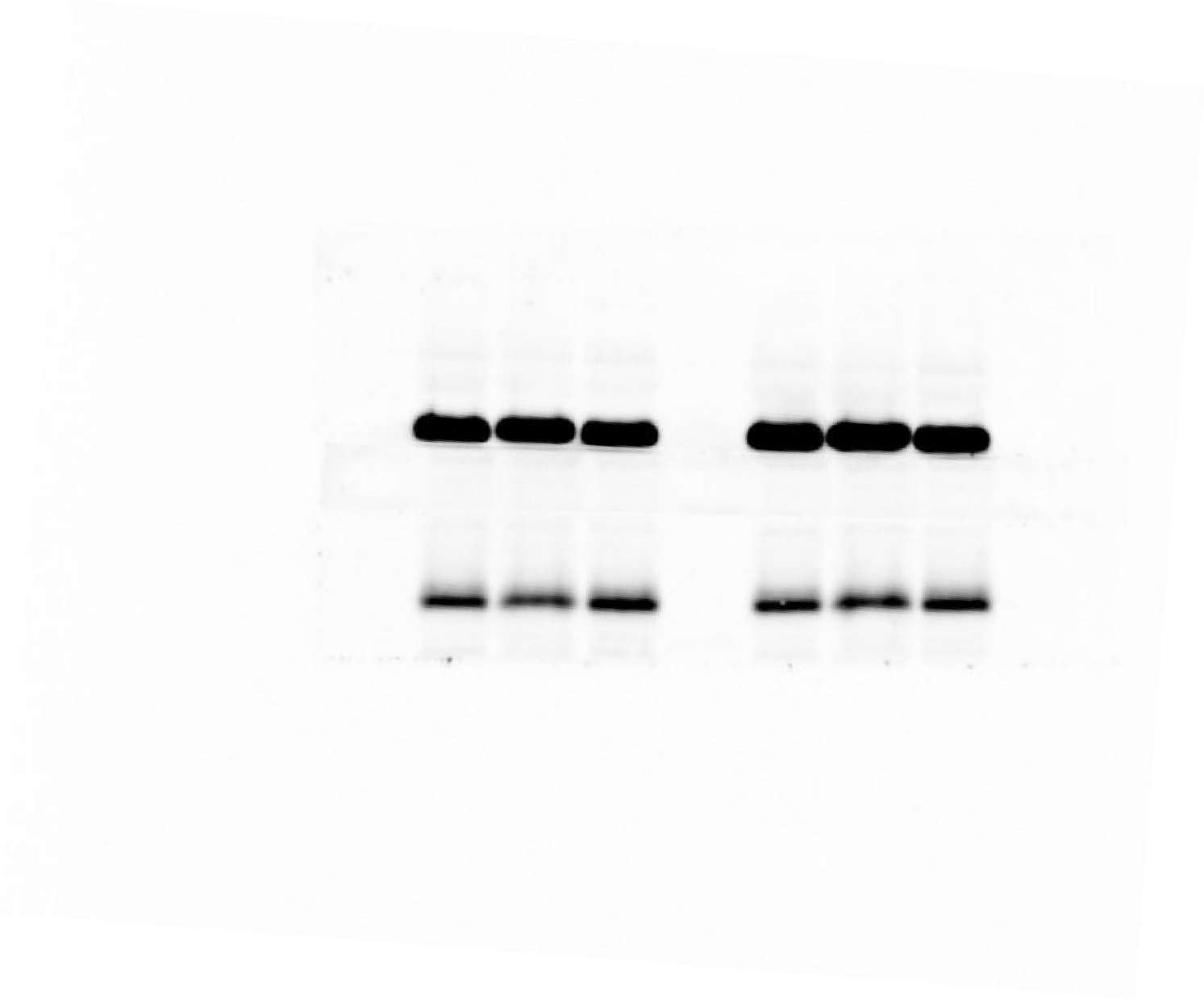

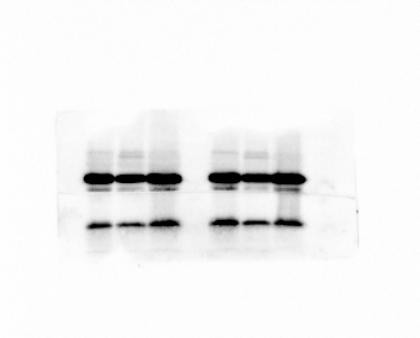

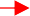

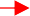

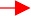

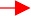


GAPDH

130KD

95KD

72KD

55KD

40KD

33KD

25KD

GPX4

15KD

GAPDH

130KD

95KD

72KD

55KD

40KD

33KD

25KD

GPX4

15KD


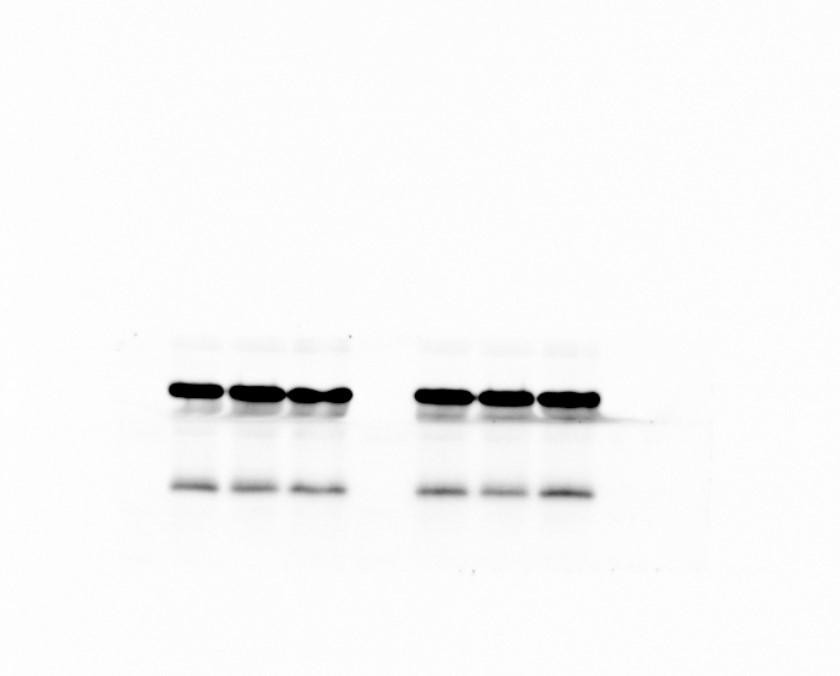

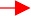

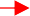


GAPDH

130KD

95KD

72KD

55KD

40KD

33KD

25KD

GPX4

15KD

## FigureS6 ——Tf


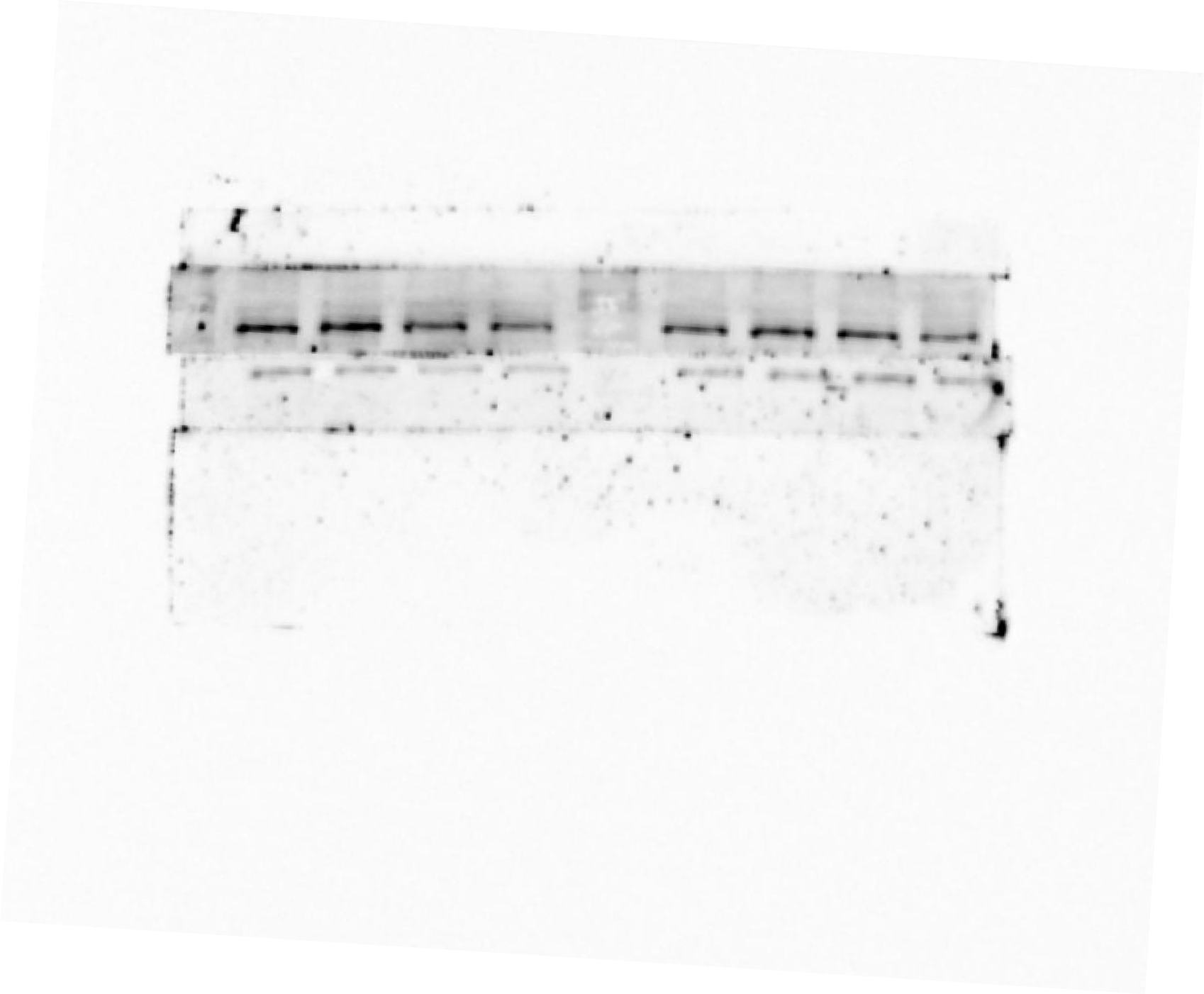

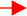

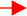


Tf

GAPDH

130KD

95KD

72KD

55KD

40KD

33KD

25KD

15KD

10KD

Tf GAPDH

130KD

95KD

72KD

55KD

40KD

33KD

25KD

15KD

10KD


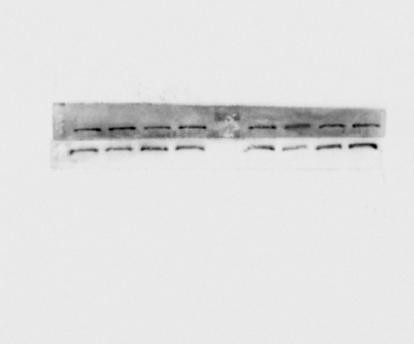

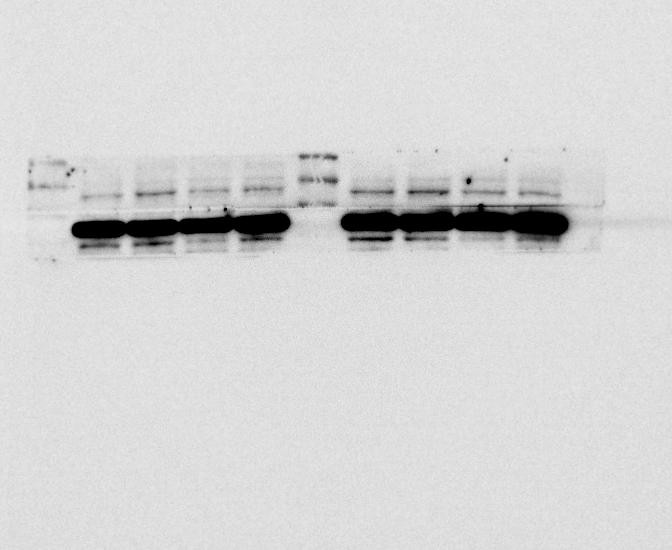

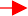

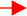

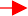

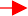


Tf GAPDH

130KD

95KD

72KD

55KD

40KD

33KD

25KD

15KD

10KD

**FigureS6 ——FTH1**


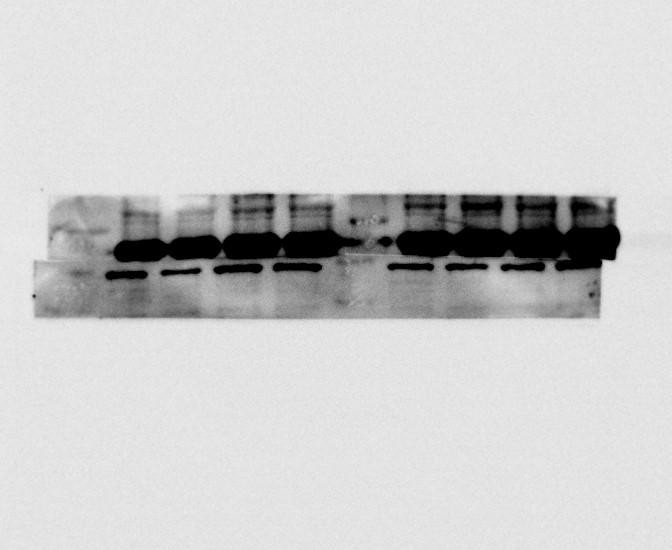

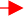


130KD

95KD

72KD

55KD

40KD

GAPDH

FTH1

33KD

25KD

15KD

130KD

95KD

72KD

55KD

40KD

GAPDH

FTH1

33KD

25KD

15KD

GAPDH

95KD

72KD

55KD

40KD

FTH1

33KD

25KD

15KD

**FigureS6——GPX4**

130KD

95KD

72KD

55KD

GAPDH

40KD

33KD

25KD

GPX4

15KD

10KD

130KD

95KD

72KD

55KD

GAPDH

40KD

33KD

25KD

GPX4

15KD

10KD

130KD

95KD

72KD

55KD

GAPDH

40KD

33KD

25KD

GPX4

15KD

10KD

**FigureS7 ——Tf**

Tf GAPDH

130KD

95KD

72KD

55KD

40KD

33KD

25KD

15KD

Tf GAPDH

130KD

95KD

72KD

55KD

40KD

33KD

25KD

15KD

10KD

10KD

Tf

GAPDH

130KD

95KD

72KD

55KD

40KD

33KD

25KD

15KD

10KD

**FigureS7 ——FTH1**

GAPDH FTH1

95KD

72KD

55KD

40KD

33KD

25KD

15KD

GAPDH FTH1

95KD

72KD

55KD

40KD

33KD

25KD

15KD

95KD

72KD

55KD

GAPDH FTH1

40KD

33KD

25KD

15KD

## FigureS7——GPX4

| 130KD  95KD  72KD  55KD |  | 130KD  95KD  72KD  55KD |
| --- | --- | --- |
| 40KD | GAPDH | 40KD |
| GAPDH 33KD |  | 33KD |
| 25KD |  | 25KD |

GPX4

15KD

GPX4

15KD

130KD

95KD

72KD

55KD

GAPDH

40KD

33KD

25KD

GPX4

15KD

## FigureS9 ——Tf

Tf

GAPDH

95KD

72KD

55KD

40KD

33KD

25KD

15KD

10KD

Tf

GAPDH

95KD

72KD

55KD

40KD

33KD

25KD

15KD

10KD

Tf

GAPDH

95KD

72KD

55KD

40KD

33KD

25KD

15KD

10KD

130KD

95KD

72KD

55KD

40KD

GAPDH

FTH1

33KD

25KD

15KD

130KD

95KD

72KD

55KD

130KD

95KD

72KD

55KD

40KD

40KD

GAPDH

FTH1

GAPDH

FTH1

33KD

33KD

25KD

25KD

15KD

15KD

GAPDH

GPX4

130KD

95KD

72KD

55KD

40KD

33KD

25KD

15KD

GAPDH

GPX4

130KD

95KD

72KD

55KD

40KD

33KD

25KD

15KD

GAPDH

130KD

95KD

72KD

55KD

40KD

33KD

25KD

GPX4

15KD

## FigureS10 ——Tf

Tf GAPDH

95KD

72KD

55KD

40KD

33KD

25KD

15KD

10KD

Tf GAPDH

95KD

72KD

55KD

40KD

33KD

25KD

15KD

10KD

Tf GAPDH

95KD

72KD

55KD

40KD

33KD

25KD

15KD

10KD

130KD

95KD

72KD

55KD

GAPDH

FTH1

40KD

33KD

25KD

15KD

130KD

95KD

72KD

55KD

GAPDH

FTH1

40KD

33KD

25KD

15KD

130KD

95KD

72KD

55KD

GAPDH

FTH1

40KD

33KD

25KD

15KD

GAPDH

130KD

95KD

72KD

55KD

40KD

33KD

25KD

GPX4

15KD

10KD

130KD

95KD

72KD

55KD

40KD

GAPDH

33KD

25KD

GPX4

15KD

10KD

GAPDH

130KD

95KD

72KD

55KD

40KD

33KD

25KD

GPX4

15KD

10KD

# Figure 5E

Control

H_2_O_2_ H_2_O_2_+Buyrate

Control H_2_O_2_ H_2_0_2_+sh LCN2

Control H_2_O_2_ H_2_O_2_+ Buyrate H_2_O_2_+ Buyrate+OE-LCN2

Control H_2_O_2_ H_2_O_2_+FP SUP H_2_O_2_+live SUP

# Figure 3G

Control H_2_O_2_ H_2_O_2_+Buyrate

# Figure 5I

Control

H2O2 H_2_O_2_+sh LCN2

# Figure 5K

Control H_2_O_2_ H_2_O_2_+ Buyrate H_2_O_2_+ Buyrate+OE-LCN2

Aging+Veh Aging+Fer1

Young-FMT Aging-FMT

Control Aging Aging+FP

Aging+Veh Aging+Butyrate

Aging Control FP△BCoAT FP△BCoAT+Butyrate

Supplement Figure3B

Healthy control Elderly HF Elderly HF+Fer-1
