# Supplementary material for: Faecalibacterium prausnitzii prevents age-related heart failure by suppressing ferroptosis in cardiomyocytes through butyrate-mediated LCN2 regulation
Source: Gut Microbes. 2025 May 13;17(1):2505119. doi: 10.1080/19490976.2025.2505119 (PMC12080280; doi:10.1080/19490976.2025.2505119)
Supplement: Supplemental Material [file KGMI_A_2505119_SM8129.zip › supplementary_material clean.docx]

**Supplemental Materials**

**Supplemental Table 1. Clinical characteristics of stool samples donors**

|  | **Elderly healthy (n=40)** | **Elderly HF (n=40)** | ***P* value** |
| --- | --- | --- | --- |
| Age, y | 70.9 ± 3.2 | 71.9 ± 4.2 | 0.108 |
| Gender, m/f | 15/25 | 19/21 | 0.497 |
| EF,(%) | 66.3 ± 5.6 | 50.9 ± 12.7 | <0.001 |
| Cholesterol,(mmol/L) | 4.2 ± 0.8 | 4 ± 1.1 | 0.336 |
| Triglyceride,(mmol/L) | 1.7 ± 0.9 | 1.3 ± 0.8 | 0.058 |
| Creatinine,(umol/L) | 80.9 ± 28.7 | 100.6 ± 71.6 | 0.042 |
| Diabetes,n(%) | 10 (25) | 11 (27) | >0.999 |
| Current smokers, n(%) | 12 (30) | 10 (25) | 0.802 |


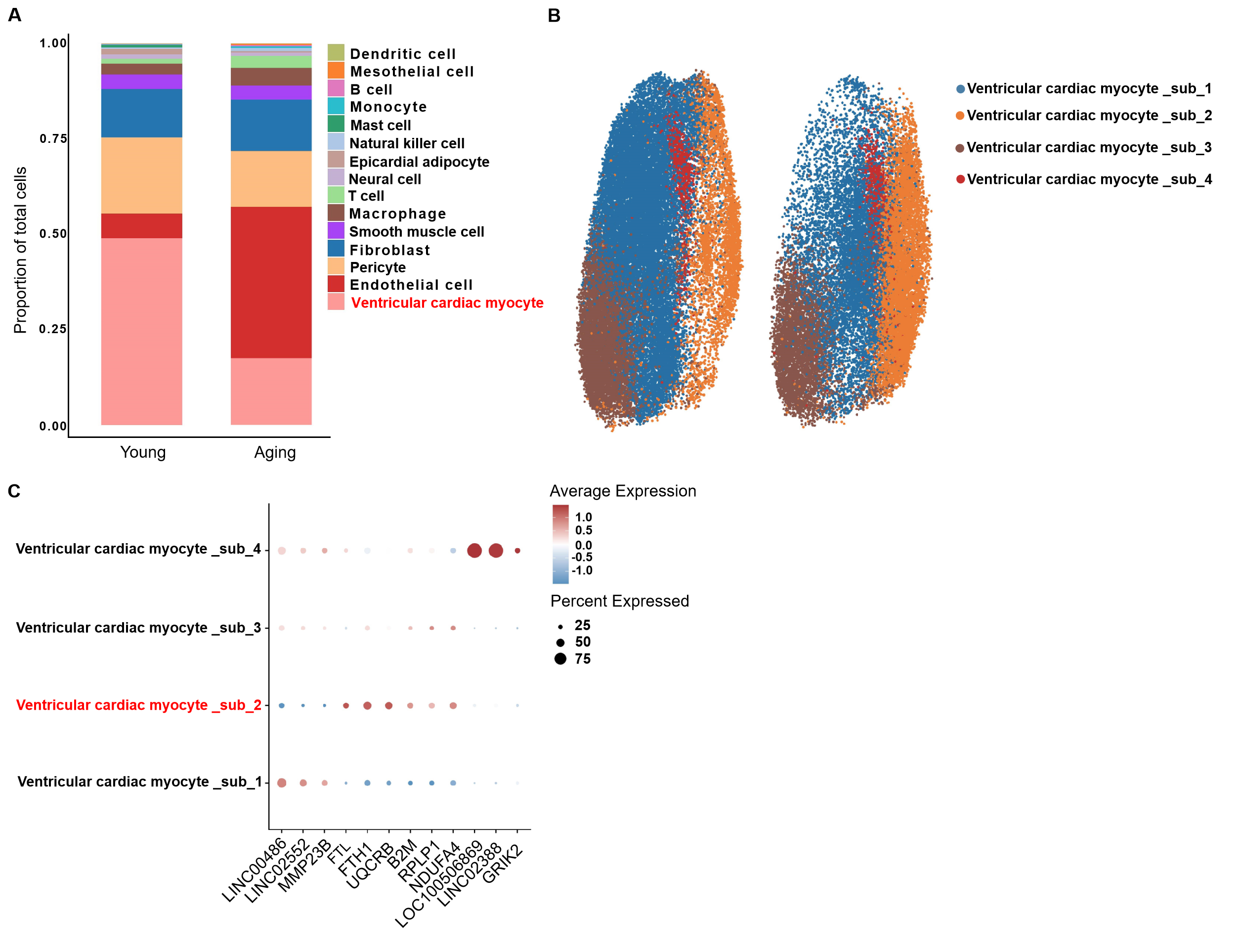


**Figure. S1 Single-cell sequencing analysis of heart tissue in Young and Aging individuals.**

**(A)** Proportional distribution of cell types in young and aging hearts.

**(B)** UMAP clustering of ventricular cardiac myocytes, revealing four distinct subtypes (sub_1 to sub_4), with each subtype represented in different colors.

**(C)** Dot plot of marker gene expression for each of the four ventricular cardiac myocyte subtypes, displaying both average expression levels and the percent of cells expressing each marker.


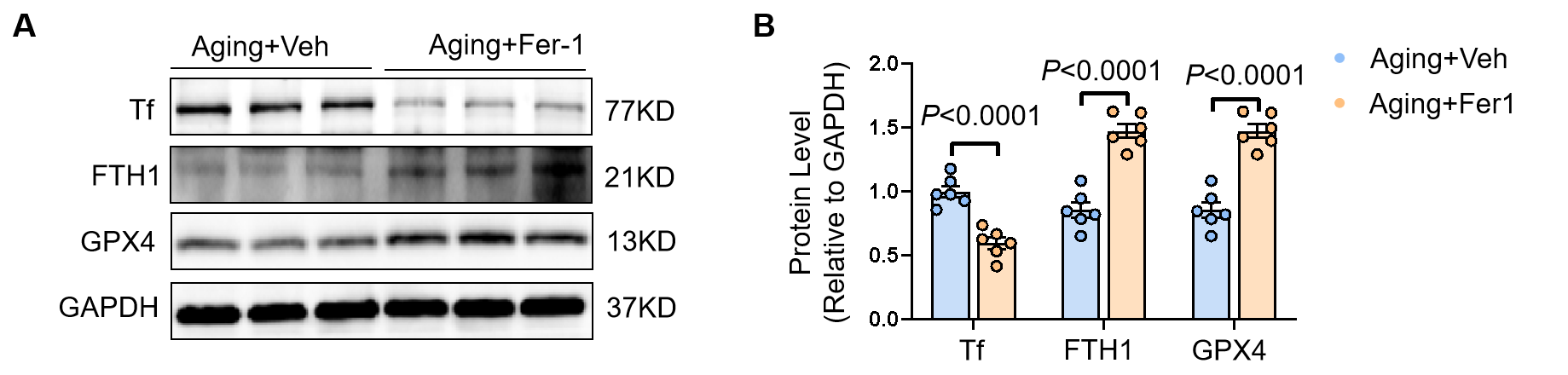


**Figure. S2 Ferroptosis inhibitors reduce ferroptosis levels in the atria of aged rats.**

1. Representative bands showing the expression of Tf, FTH1 and GPX4 in the heart of rats from Aging+Veh and Aging+Fer-1 groups.
2. Quantification of expressions of Tf, FTH1 and GPX4 in the heart of rats from Aging+Veh and Aging+Fer-1 groups (n=6 per group).

The data are given as mean ± SEM and compared by Student’s t test.


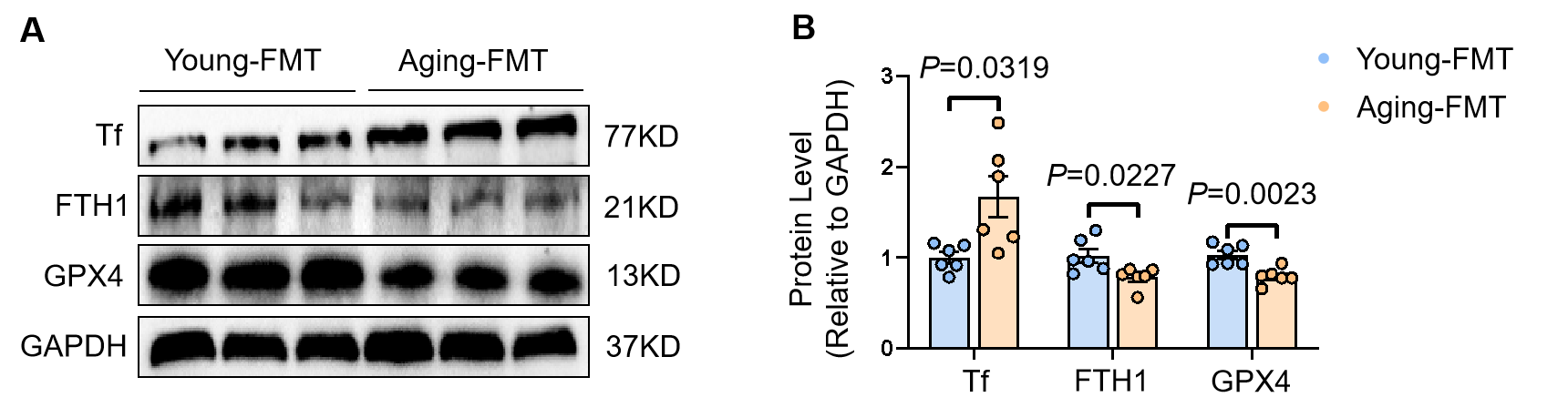


**Figure. S3 Gut microbiota transplantation from aged rats exacerbates cardiac ferroptosis in recipient rats.**

1. Representative bands showing the expressions of Tf, FTH1 and GPX4 in the hearts of rats from Young-FMT group and Aging-FMT group.
2. Quantification of Tf, FTH1 and GPX4 in the hearts of rats from Young-FMT group and Aging-FMT group (n = 6 per group).

The data are given as mean ± SEM and compared by Student’s t test.


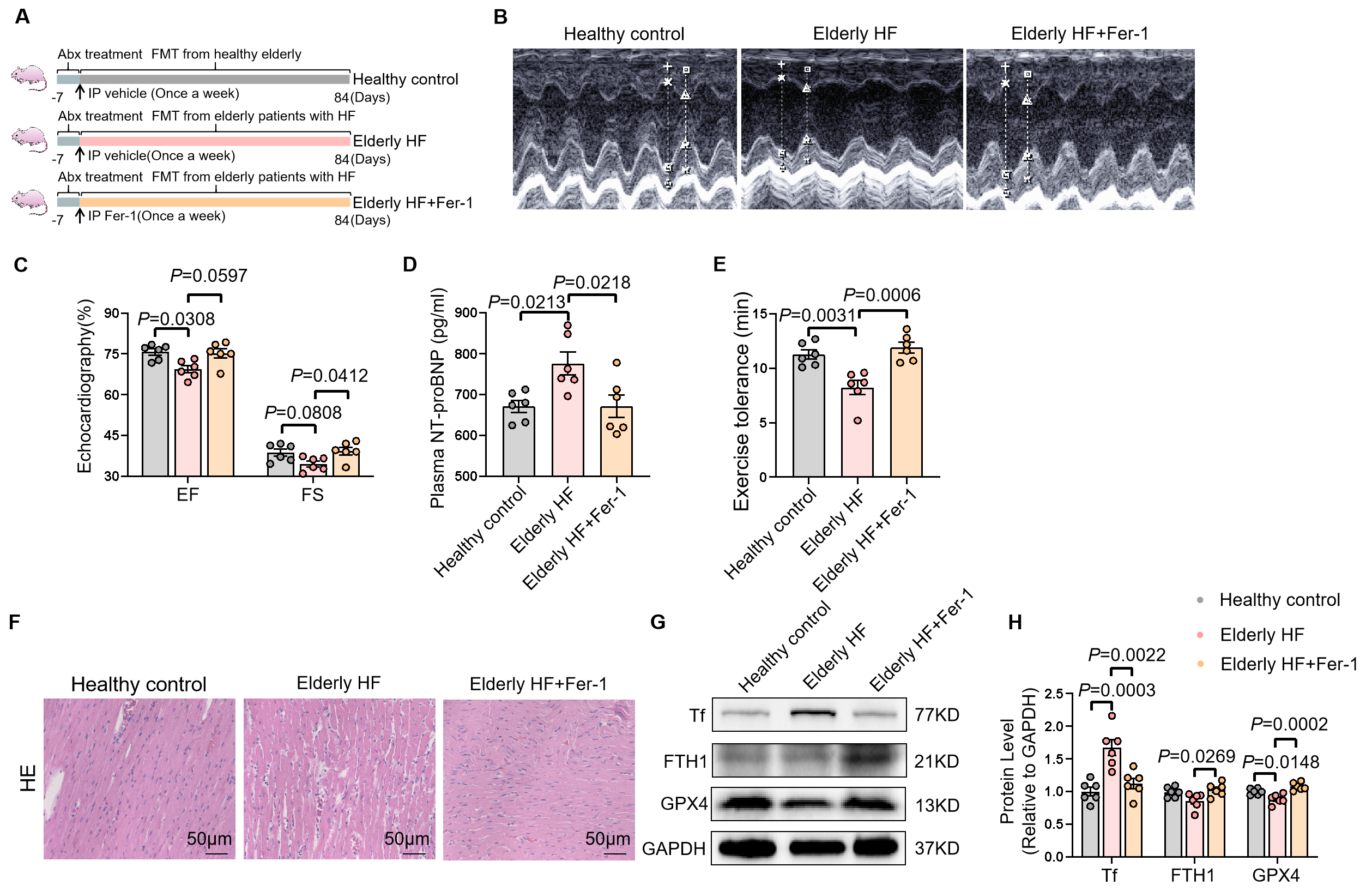


**Figure. S4 Ferroptosis inhibitor reduces heart ferroptosis in recipient rats induced by fecal microbiota transplantation from elderly heart failure patients.**

1. Schematic illustration of the experimental design for ferroptosis inhibitor intervention. Before microbial transplantation, rats were treated with antibiotics for 1 week to deplete gut microbiota. Rats were subjected to receive microbial transplantation of healthy controls, elderly HF patients, and elderly HF patients combined with ferroptosis inhibitor (ferrostatin-1, 0.8mg/kg, intraperitoneal injection), once a week for 12 weeks.
2. Representative M-mode images of left ventricular wall motion in the hearts.
3. Statistical data of cardiac ejection fraction (EF) and fraction shortening (FS) of rats (n=6 per group).
4. Quantitative analysis of plasma NT-proBNP levels in rats (n=6 per group).
5. The exercise tolerance in rats (n=6 per group).
6. Representative images of HE staining of the left ventricle of hearts in rats.
7. Representative bands showing the expressions of Tf, FTH1 and GPX4 in the heart of rats (n = 6 per group).
8. Quantification of Tf, FTH1 and GPX4 in the heart of rats (n = 6 per group).

The data are given as mean ± SEM and compared by One Way ANOVA.


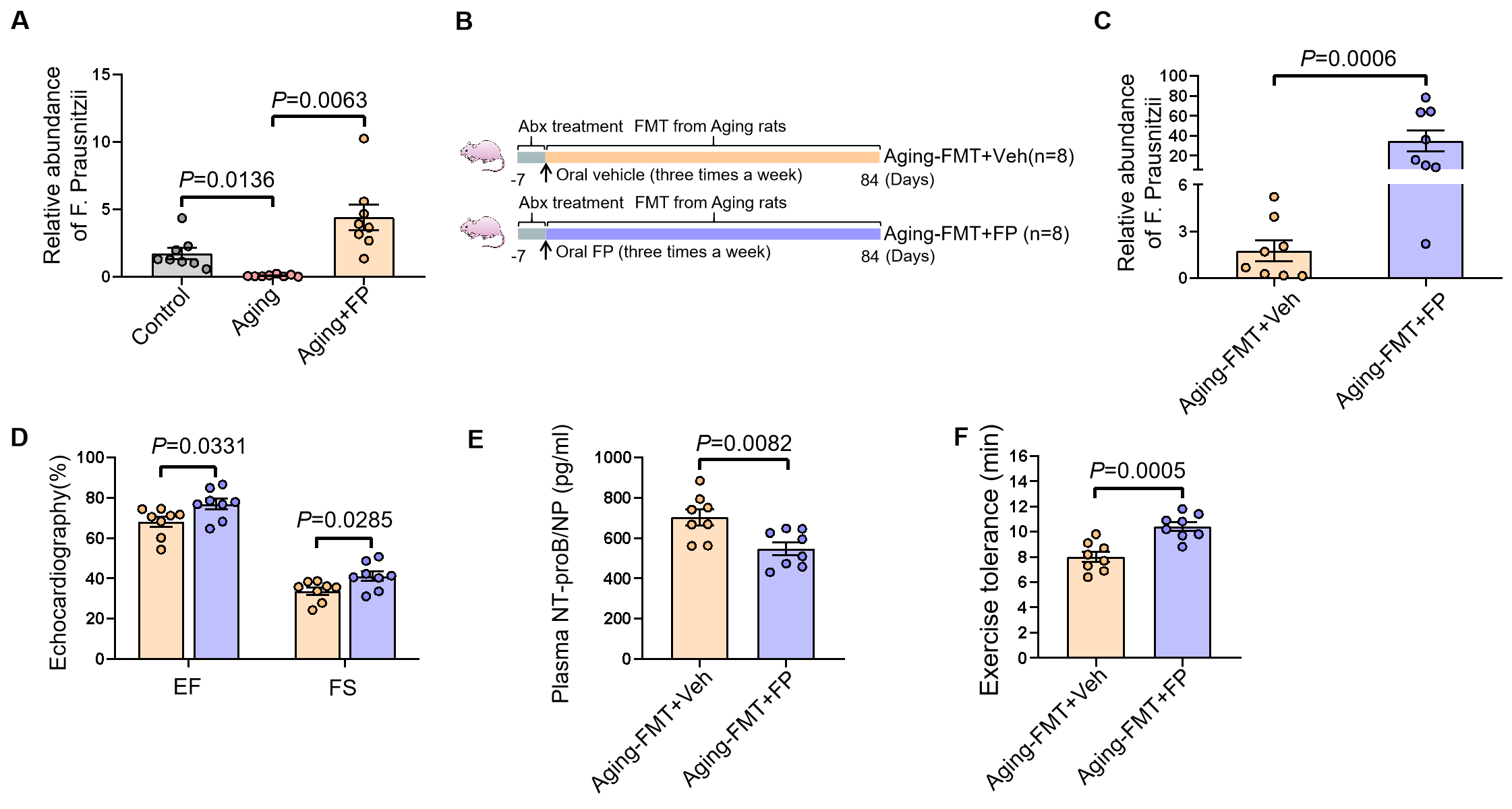


**Figure. S5 Oral** **supplementation of** ***F. prausnitzii* prevents cardiac dysfunction in rats induced by the transplantation of aged microbiota.**

1. The relative abundance of *F. prausnitzii* in rats (n=8 per group).
2. Schematic illustration of the experimental design for *F. prausnitzii* supplementation. Before microbial transplantation, rats were treated with antibiotics for 1 week to deplete gut microbiota. Rats received aged microbial transplantation were randomly divided into two groups, one group received vehicle, another group received *F. prausnitzii* gavage, once a week for 12 weeks.
3. The relative abundance of *F. prausnitzii* in rats (n=8 per group).
4. The statistical data of cardiac ejection fraction (EF) and fraction shortening (FS) in rats (n=8 per group).
5. The levels of NT-proBNP in plasma from Aging-FMT+veh group and Aging-FMT+Fp group (n=8 per group).
6. The exercise tolerance in rats (n=6 per group).

The data are given as mean ± SEM and compared by Student’s t test.


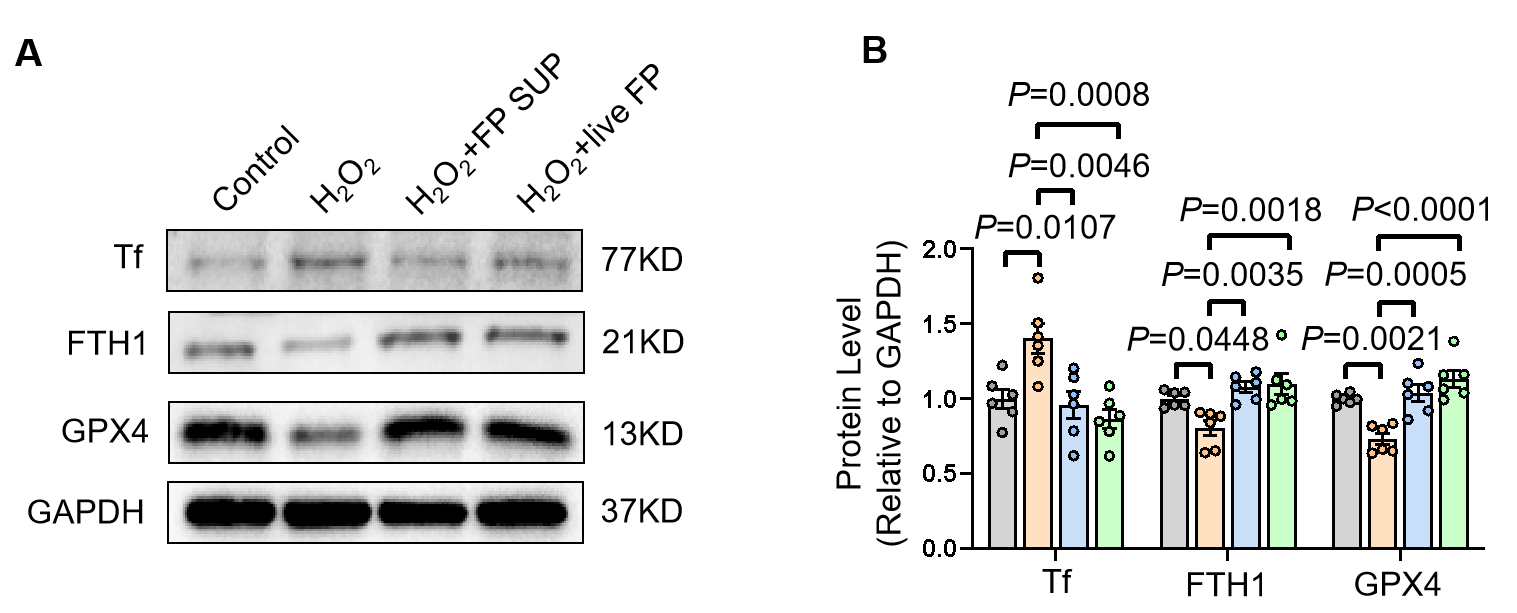


**Figure. S6 Live *Faecalibacterium prausnitzii* and its supernatant alleviate H_2_O_2_-induced ferroptosis in cardiomyocytes.**

**(A)** Representative bands showing the expressions of Tf, FTH1 and GPX4 in the cardiomyocytes from each groups.

**(B)**Quantification of Tf, FTH1 and GPX4 in the cardiomyocytes from each groups (n = 6 per group).

The data are given as mean ± SEM and compared by One Way ANOVA.


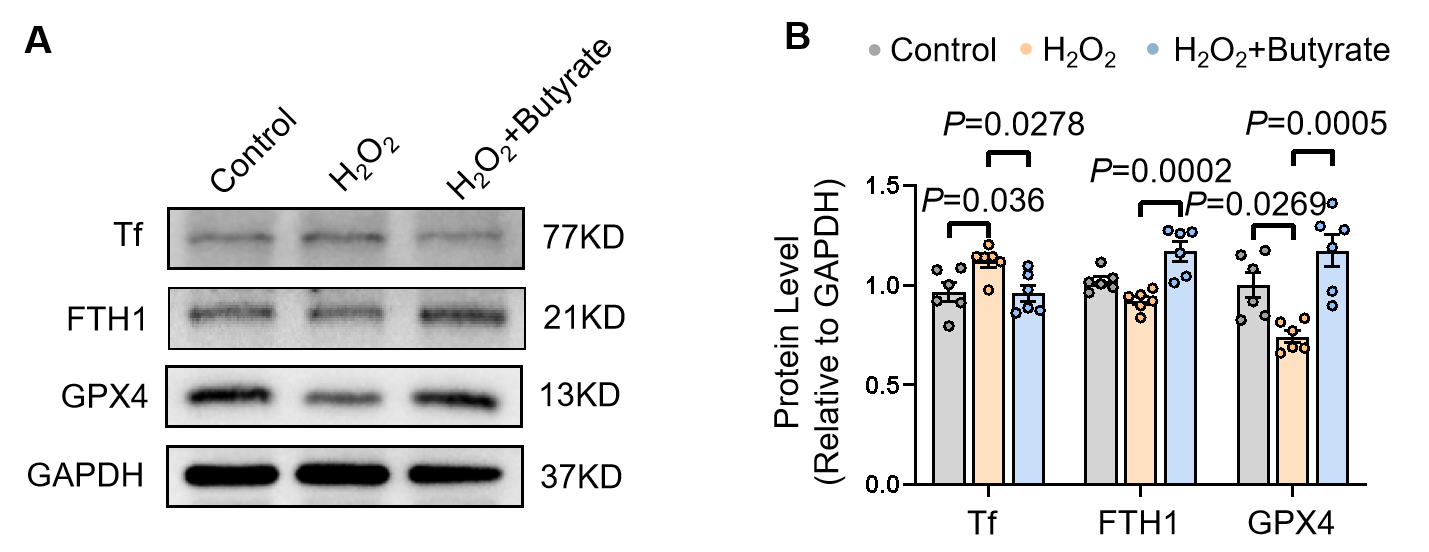


**Figure. S7 Butyrate alleviates H_2_O_2_-induced ferroptosis in cardiomyocytes.**

**(A)** Representative bands showing the expressions of Tf, FTH1 and GPX4 in the cardiomyocytes from each groups.

**(B)**Quantification of Tf, FTH1 and GPX4 in the cardiomyocytes from each groups (n = 6 per group).

The data are given as mean ± SEM and compared by One Way ANOVA.


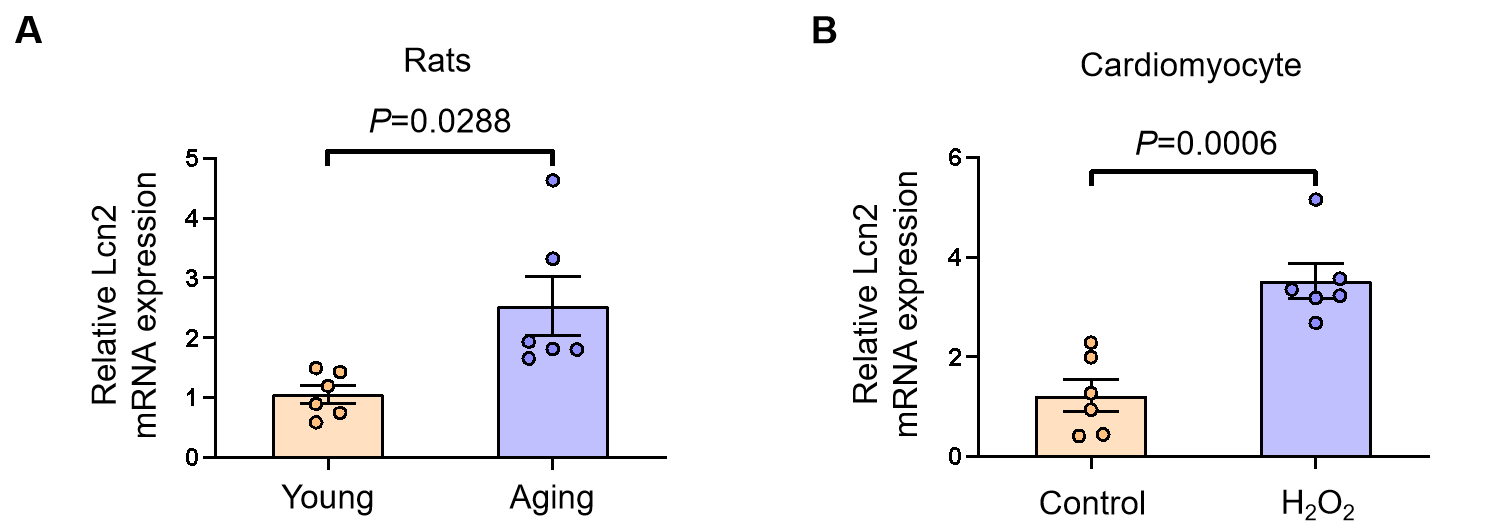


**Figure. S8 The relative expression of LCN2 in rats and** **cardiomyocyte.**

**(A)**The relative expression of LCN2 in the left ventricle of heart in rats (n=6 per group).

**(B)** The relative expression of LCN2 in cardiomyocytes (n=6 per group).

The data are given as mean ± SEM and compared by Student’s t test.

.


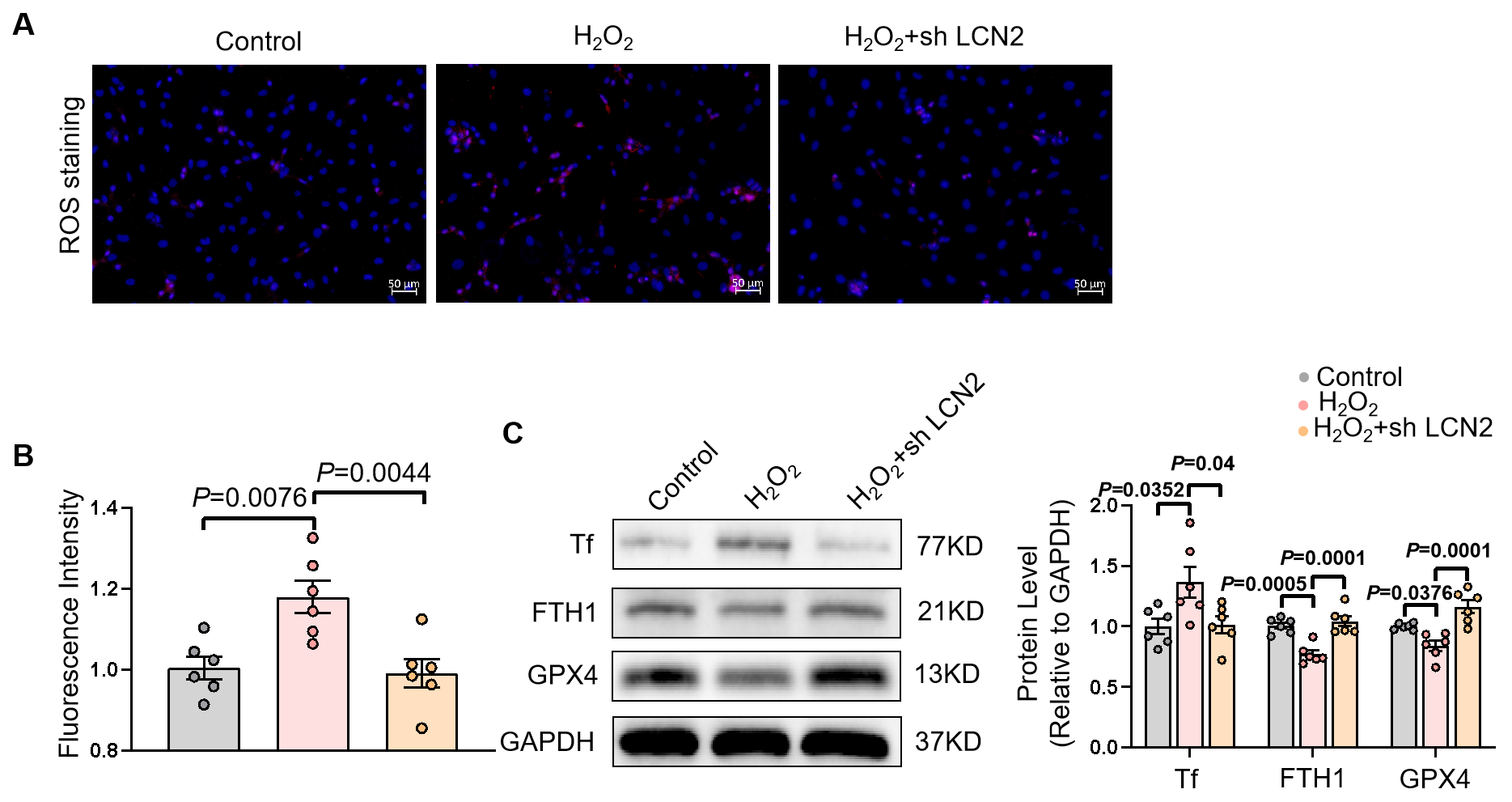


**Figure. S9 Silencing LCN2 reduces reactive oxygen species levels in senescent cardiomyocytes and alleviates ferroptosis.**

1. Representative ROS staining images of cardiomyocytes.
2. The fluorescence intensity of ROS in cardiomyocytes in each groups (n=6 per group).
3. Representative bands showing the expressions of Tf, FTH1 and GPX4 in the cardiomyocytes from each groups.
4. Quantification of Tf, FTH1 and GPX4 in the cardiomyocytes from each groups (n = 6 per group).

The data are given as mean ± SEM and compared by One Way ANOVA.


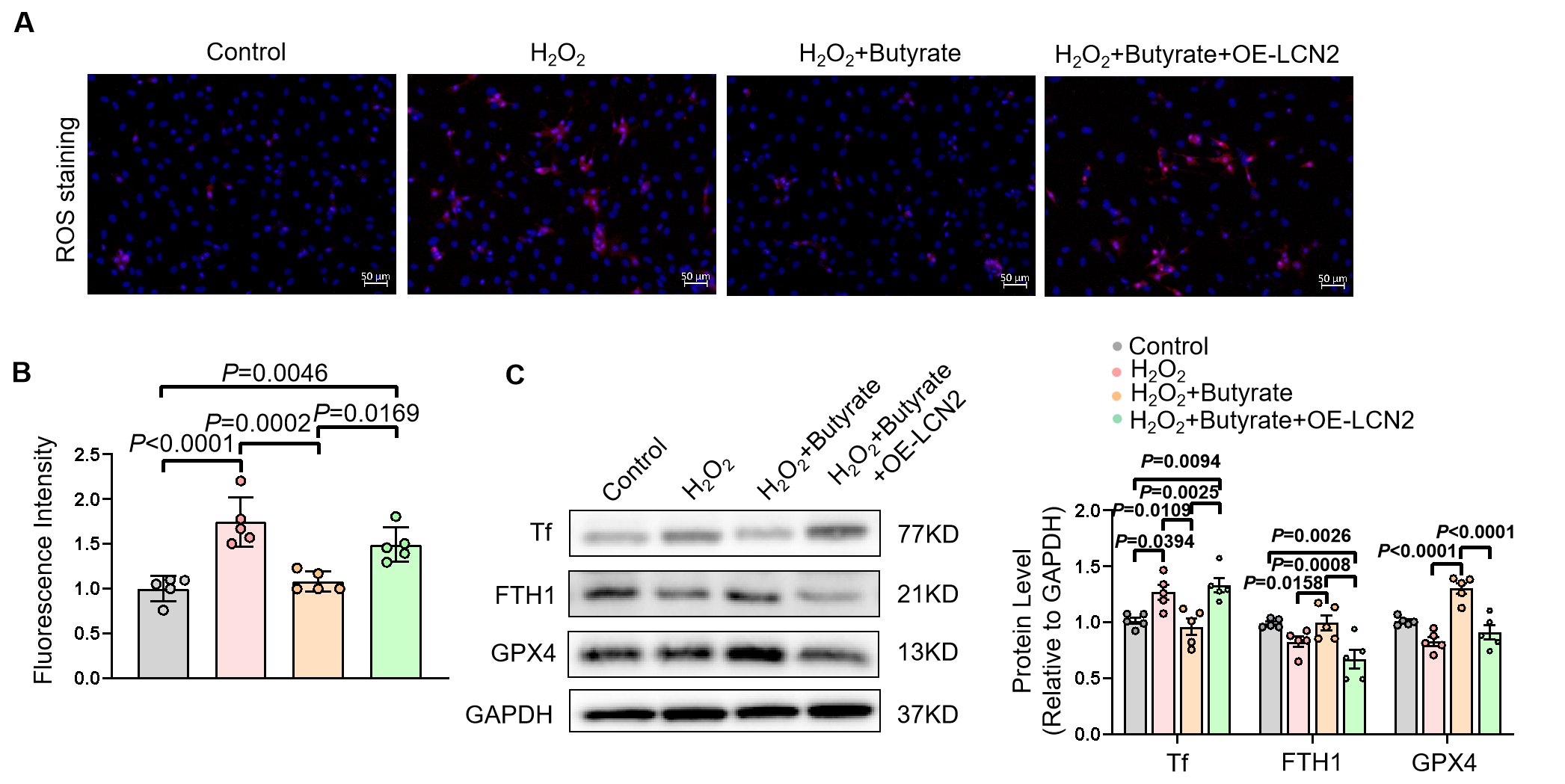


**Figure. S10 Overexpression of LCN2 counteracts the protective effect of butyrate against ferroptosis in cardiomyocytes.**

1. Representative ROS staining images of cardiomyocytes.
2. The fluorescence intensity of ROS in cardiomyocytes in each groups (n=6 per group).
3. Representative bands showing the expressions of Tf, FTH1 and GPX4 in the cardiomyocytes from each groups.
4. Quantification of Tf, FTH1 and GPX4 in the cardiomyocytes from each groups (n = 6 per group).

The data are given as mean ± SEM and compared by One Way ANOVA.
